# A bivariate multifractal analysis approach to understanding socio-spatial segregation dynamics

**DOI:** 10.1038/s41598-025-86024-9

**Published:** 2025-02-07

**Authors:** Janka Lengyel, Stéphane G. Roux, Olivier Bonin, Stéphane Jaffard, Patrice Abry

**Affiliations:** 1 CNRS, LPENSL, UMR5672, ENS de Lyon, 69342 Lyon cedex 07, France; 2 École nationale des ponts et chaussées, LVMT, Univ. Gustave Eiffel, 77420 Champs-sur-Marne, France; 3 LAMA, Univ. Paris-Est Créteil, 94010 Créteil, France; 4 School of Mathematics and Science, Institute of Physics, ForWind - Center for Wind Energy Research, Carl von Ossietzky Universität Oldenburg, 26129 Oldenburg, Germany

**Keywords:** Applied mathematics, Statistical physics, Socioeconomic scenarios, Nonlinear phenomena

## Abstract

Although the study of multifractal properties is now an established approach for the statistical analysis of urban data, the joint multifractal analysis of several spatial signals remains largely unexplored. The latter is crucial for understanding complex multiscale relationships in cities, such as socio-spatial segregation processes, where the evolution of behavior across geographical scales traditionally plays a central role. In this context, the proposed approach, which uses wavelet leaders for multifractal analysis of irregular point processes, estimates self-similarity and intermittency exponents as well as self-similar and multifractal cross-correlation by combining classical multifractal and geographic analysis methods. Results show that a local bivariate multifractal analysis can not only be related to classical two-group segregation indices but also extends them to provide a robust analytical framework that *(1)* is less susceptible to the modifiable areal unit problem and normalization methods and that *(2)* can reveal more pronounced evolution across spatial scales. In addition, multifractal analysis *(3)* can also delineate more “perturbed” areas in which the dominance of one group is occasionally interrupted by local concentrations of the other group, referred to here as intermittent segregation.

## Introduction

### Residential segregation and the role of geographical scales

In its broadest sense, residential segregation refers to the degree to which various population groups inhabit or encounter distinct social surroundings^1^. Because it is one of the most fundamental processes in human geography, consistent and correct measurement of segregation is critical^2^; most experts agree that spatial segregation is a complex attribute of an urban system, difficult to capture with a single index^3^. There are, therefore, numerous classical and well-established measures; for a comprehensive overview, see, e.g.^1,2,4,5^. Massey and Denton^5^ initially assigned these to five dimensions—*evenness, exposure, clustering, centralization, and concentration*—which were later combined by Reardon and O’Sullivan^1^ into two; evenness and exposure. Along the *evenness* axis, which examines the differential distribution of population components, the most widely used are the dissimilarity index^2,6–9^ and its generalized version^10^, together with numerous entropy-based indices, e.g., the diversity index^11,12^ and the H-index from spatial information theory^1,4,13^. On the second *exposure* axis, which refers to the extent to which individuals of one group come into contact with individuals of another group^1^, the isolation and exposure indices are the most widespread^2,5,14^. Moreover, the importance of fluctuations in residential segregation (1) within a given city or urban system and (2) across spatial scales is now widely recognized. In the context of (1) *intra-urban variation*, conventional indices have been criticized^15^ for their global character, as they attempt to represent an entire city with a single index while ignoring the spatial arrangement of population distribution, also known as the “checkerboard problem”^6^; They have therefore been extended to include additional spatial and local measures of segregation^14,16^. Attempts towards (2) *multiple level descriptions*^12,13,15,17,18^ or examining how segregation evolves across geographical scales have also been made extensively, and aspects of this are now discussed in more detail in the following subsections of this review.

*Multilevel description and sensitivity to geographical boundaries. * Since we rarely have access to desegregated data, the problem of modifiable area units (MAUP) raises issues in segregation analysis, as is also the case in other geographical research areas. For anonymity purposes, small-scale data are often limited to administrative subdivisions such as census tracts or specific neighborhood boundaries, and these subdivisions may have different resolutions across urban systems, making relevant comparisons difficult. As has often been noted, administrative units tend to be aligned by jurisdiction or historical roots and often do not capture the inherent spatial processes in the data^17^. Similarly, analysis at the level of administrative boundaries can lead to a discrepancy between the scale of analysis and the characteristic scale of the phenomenon^17^. Moreover, the computation of currently existing local and spatial segregation indices is often based on a global-local comparison approach between two or more selected/available administrative boundaries, where the local resolution may evolve across scales to obtain a multilevel description. The intuition behind this is the assumption that in a non-segregated system, the local distribution of subpopulation groups will broadly coincide with the overall global distribution^16,19^. Furthermore, spatialized versions of classical segregation indices^15,17,20^ have been designed to account for local spatial configurations but ultimately yield a global index that poses numerous challenges in terms of interpretability and sensitivity to normalization techniques. Finally, and most importantly, multiscale segregation indices have so far worked independently, scale by scale, without taking into account the interaction and interdependencies between neighboring spatial resolutions.

### Multifractal analysis

Multifractal analysis, which describes the properties of fluctuations of local regularity in time or space, is now regarded as a tested technique for statistical image analysis^21^ and has been extensively applied in various fields, including medical research^22^ and satellite image processing^23^. In the context of geographical analysis, the fundamental idea is to explore the spatial complexity and organization in cities, polycentric settings, and urban-rural systems. This investigation relates to their morphological structures^24,25^, societal aspects^26,27^, and economic frameworks^27–30^. Instead of traditional geographical concepts, the analysis employs scaling exponents to capture the multiscale interdependencies of the latter and to try to uncover qualitatively new characteristics of urban structures. Until now, however, the investigation into the multifractality of sociospatial processes has seen limited application within the context of segregation. There have been a few noteworthy exceptions in the form of univariate studies. For instance, one approach involves calculating Renyi’s generalized dimensions for racial patterns using the box-counting method^3^ (note that the extension of this context to the bivariate setting has been sketched in^31^ which is the seminal article on which multivariate multifractal analaysis is based). More broadly, some studies linked socioeconomic inequalities to the multifractality of urban structures, e.g., using the example of housing prices^27,28,30^.

**Fig. 1:**
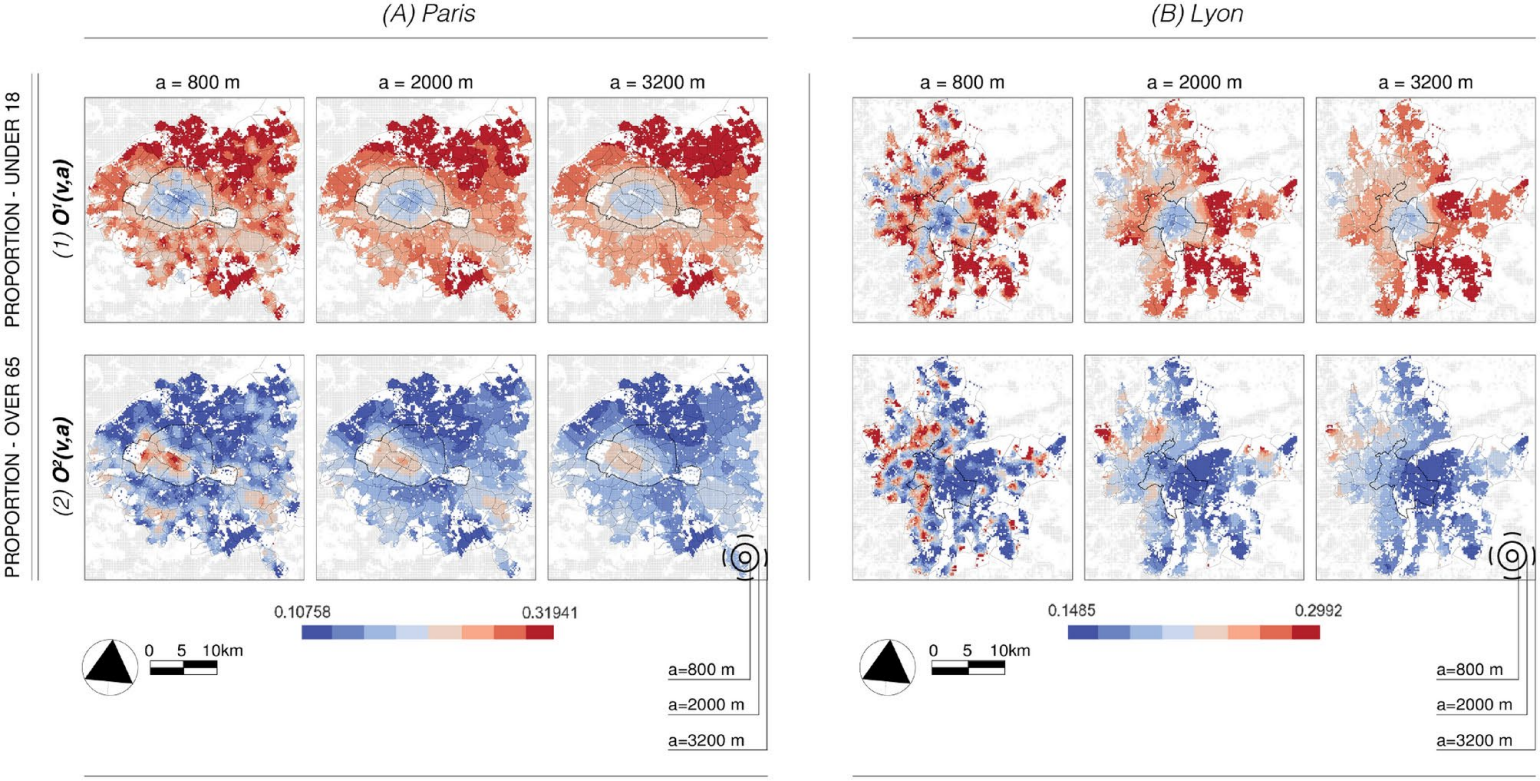
*The two original data show the demographic structure.* The weighted mean $O^i(v,a)$ at three spatial scales: the finest, middle and largest observed length scales *a* for the proportion of the population under 18 ($i=1$) and over 65 ($i=2$). The population over 65 is concentrated in the center of Paris (**A**), while the outer districts-predominantly the northern ones-have a high proportion of people under 18. In Lyon (**B**), the aging population is clearly present along the western periphery, while the younger population settles in all suburbs, but mainly east of Lyon.

Recently, a new mathematical framework has emerged that provides a solid theoretical foundation and practical effectiveness for conducting multivariate multifractal analyses^21,32,33^ for data defined on regular grids; its main goal is to detect cross-correlations between the multiscale local fluctuations of the different data components. Independently, a systematic approach has emerged that is carefully designed to perform univariate multifractal analysis in sparse spatial point processes in arbitrary local environments^25,26,29,30^. This paper builds on these two approaches to examine two-group urban segregation. It must be emphasized, however, that although, as described above, the use of univariate (multi)fractal analysis has been explored in urban research, the development of the bivariate methodology in a non-regular sampling setting has not been performed before, and is one of the main contributions of this study. A detailed discussion of this approach is presented in the methodology section.

### Goals

The aim of this study is to conduct a multiscale analysis of socio-spatial segregation using indicators with the following characteristics in order to overcome some of the limitations of classical segregation measures as well as to extend them.

1. *Unaffected by zoning and global boundary. * Given the challenges described above, it remains necessary to develop socio-spatial segregation analysis techniques that are free of administrative boundaries^20^ at both local and global levels and ensure relative consistency of analyses at different scales and case studies^12^.
2. *Dynamic multiscale approach. * Secondly, we introduce a more dynamic perspective into the evenness/exposure dimensions of segregation research by emphasizing the *fluctuations* in the distribution of population groups within their immediate local neighborhoods, i.e., the wavelet technique. These variables are then incorporated into a truly multiscale approach, i.e., the bivariate multifractal analysis.
3. *Local non-homogeneity and characteristic scales. * This procedure allows to reveal more detailed multilevel information and variations *within the observed territories* and define characteristic scales for the analysis.
4. *Intermittent segregation. * Alongside this *combined local, spatial, and multiscale* outlook on segregation measures, we propose a novel approach to describing highly unusual dynamics of spatial interactions between two population groups where the dominance of one group is occasionally interrupted by local concentrations of the other.

## Data and case study

We use high-resolution French census data from the French National Institute of Statistics and Economic Studies (INSEE^34^), available on a $200 \times 200$ m grid for 2015. The first study area in Fig. 1.A is the city of Paris in France, and its immediate surroundings, the so-called *Petite Couronne*. The second area comprises Lyon, the third largest city in terms of population in France, and its first urban ring. The delimited area in Fig. 1.B corresponds to the metropolitan area of Lyon as defined by INSEE^34^, but without the spatially separated southern city of Givors in order to* (1)* keep the two study areas approximately the same size and *(2)* to maintain a continuous urban structure as much as possible. The total extent of the areas investigated here covers roughly $36\times 36$ km around Paris (Fig. 1.A) and $32\times 32$ km around Lyon (Fig. 1.B). Because French data do not include information on ethnicity, the most commonly studied variable in research on residential segregation, we focus our analysis on demographic parameters. Demographic aspects are the proportion of people under eighteen and over sixty-five. These can also be seen as *proxies for economic inequalities*, as in the Paris region, for example, we find a strong Pearson correlation coefficient $-0.81$ between the proportion of under-18s and equivalized disposable income per capita (Table 1). In the supporting material (hereafter referred to with *S*) in Fig. S1, remarkable similarities can be observed between the distribution of the proportion of people aged over 65 and the per capita equivalized disposable income as well as between the proportion of people under 18 and the proportion of poor households.

**Table 1:** Correlations between selected INSEE variables.

PARIS region	$\%\,poor\,Hh.$	$EDI\,(p.c.)$	$\%\,Under\,18$	$\%\,Over\,65$
$\%\,poor\,Hh.$	1			
$EDI\,(p.c.)$	− 0.76	1		
$\%\,Under\,18$	0.65	− 0.81	1	
$\%\,Over\,65$	− 0.68	0.81	− 0.81	1

LYON region	$\%\,poor\,Hh.$	$EDI\,(p.c.)$	$\%\,Under\,18$	$\%\,Over\,65$
$\%\,poor\,Hh.$	1			
$EDI\,(p.c.)$	− 0.68	1		
$\%\,Under\,18$	0.24	− 0.27	1	
$\%\,Over\,65$	− 0.23	0.27	− 0.54	1

The demographic structure—the share of people under 18 and over 65—can be closely linked to socioeconomic indicators: the proportion of poor households (H.h.) and the equivalized disposable income (EDI) per capita (p.c.).

## Methodology

This section first describes the computation of the selected classical segregation measures and identifies their main challenges. The second part introduces local multifractal analysis in univariate and bivariate settings, and discusses the main differences with the methodology illustrated in the first part. It should be noted that many segregation studies rely on specific synthetic examples to demonstrate methodology. Because we are interested in both the evenness and exposure dimensions of segregation simultaneously in our study, we refrain from using synthetic examples that typically contain spatially shifted but homogeneous populations usually expressed by checkerboard structures^7,16,19^. Instead, we focus on the most commonly used indices of sociospatial segregation and compare their results with those of multifractal analysis using the same real-world datasets.

*Marked spatial point processes.*  Throughout the study, we denote by *v* the position of the local analysis and *a* the radius of the ball centered on *v* in which the indices are computed. The dataset $\mathcal{S}(v,\kappa ^i_v,\kappa ^t_v)$ is defined by its geolocation $v=(x_v, y_v)$ with $x_v$ the longitude and $y_v$ latitude coordinate (the support), along with one or more associated values $\kappa ^i_v \text { and } \kappa ^t_v$ (the marks). To ensure consistency between the two methodological sections, the superscript *t* stands for the total population, including all demographic groups between 0 and 80 and above^34^, while *i* ($i = (1,2)$) represents the respective subgroup value. Both the classical segregation measures and the multifractal parameters necessitate the computation weighting functions. We will adopt the bi-square kernel for this study since it is the most commonly used kernel in segregation research. It is obtained for any weighting distance $a'$ as

1 $$\begin{aligned} w_{v,v'} (a') = {\left\{ \begin{array}{ll} \left( 1 - \left( d_{v,v'}/a'\right) ^2\right) ^2 \text {if } d_{v,v'} < a'\\ 0 \text {, otherwise} \\ \end{array}\right. } \text {with }d_{v,v'} = \sqrt{(x_{v'} - x_v)^2 +(y_{v'} - y_v)^2 } \end{aligned}$$

where the weights are normalized so that $\sum _{v'} w_{v,v'}(a') = 1$. For any variable *X*(*v*, *a*), which jointly depends on the spatial distribution $v=(x_v, y_v)$ and the scale *a*, we define the geographically weighted mean, and variance as

2 $$\begin{aligned} \widetilde{M}_{X,a'}(v,a)=\sum _{v'} w_{v,v'}(a') X(v',a), \end{aligned}$$

3 $$\begin{aligned} \widetilde{V}_{X,a'}(v,a)=\sum _{v'} w_{v,v'}(a') \left( (X(v',a)-\widetilde{M}_{X,a'}(v,a)\right) ^2. \end{aligned}$$

The global mean is then defined as $\overline{X}(a) = \frac{1}{N_v} \sum _{v} X(v, a),$ where $N_v$ is the total number of available sites *v*. In the following parts of this article, we will use the notation $n_v (a)$ to refer to the number of available sites *v* in a ball of radius *a*. For data *X*(*v*) that only depend on space, we simply write the above-defined averages as $\widetilde{M}_{X,a'}(v)$, $\widetilde{V}_{X,a'}(v)$ and $\overline{X}$, removing the additional variable *a*.

### Classical segregation measures

As references, we selected two classical two-group segregation measures. To ensure comparability to the results of the bivariate multifractal analysis, these must be from the family of both *local* and *spatial* segregation indices. Moreover, classical segregation measures are not only sensitive to the MAUP problems described in the introduction but also to so-called *grouping systems*^15^, which, in this context, refers to the size of the subpopulations in relation to the total population, i.e. whether the analysis is conducted in a context with two or more groups. As we will show, this plays an important role in the selection of indices for this study. In the *evenness* dimension of segregation, the dissimilarity index *D*^13,15,16,18^ is by far the most commonly used index and is thus well suited for cross-comparison with other studies^15,35^. Even though it is now known that the local and spatial dissimilarity $\widetilde{D}(v,a)$ (Eq. 5) can depend strongly on the chosen global boundary, it has been shown to be more robust to grouping systems^15^ than, for example, the H-index of spatial information theory ^1,4^, which is also frequently used in the context of evenness. Given the importance of this robustness for methodological transparency in this study, we chose the dissimilarity index $\widetilde{D}(v,a)$ to measure evenness and the widely used exposure index $\widetilde{P}^{1,2}(v,a)$^1,5^ (Eq. 6) as a measure of *exposure*.

*Multiscale quantities.*  Classical segregation measures necessitate the computation of so-called local “population intensities” using a distance decay function of a certain shape. Accordingly, if $\kappa ^{t}_{v}$ and $\kappa ^i_{v}$ are the total population and the number of people belonging to subgroup *i* in census tract *v*, then their spatially smoothed values at radius *a* from every point $v(x_v,y_v)$—in accordance with Eq. (2)—are defined as $\widetilde{M}_{\kappa ^{t},a}(v)= \sum _{v'}w_{v,v'}(a) \kappa ^{t}_{v'} \text {\quad and \quad } \widetilde{M}_{\kappa ^{i}, a}(v)= \sum _{v'}w_{v,v'}(a) \kappa ^i_{v'}.$ The local ratio of the above two parameters describes the main component of both here discussed classical segregation measures according to

4 $$\begin{aligned} \widetilde{\tau }^i(v,a)= & \frac{\widetilde{M}_{\kappa ^{i}, a}(v)}{\widetilde{M}_{\kappa ^{t},a}(v)} \end{aligned}$$

*Evenness.*  If $\widetilde{\tau }^i(v,a)$ (Eq. 4) is the ratio of local population intensities or the weighted mean of the share of population groups, then in its spatial, and generalized form, the *localized* $\widetilde{D}(v,a)$ *and the global D*(*a*) *dissimilarity is defined as*

5 $$\begin{aligned} \widetilde{D}(v,a)= \sum _i \frac{\kappa _v^{t}}{2IK^{t}} |\widetilde{\tau }^i(v,a) - {\Pi }^i| \quad {\text{and}} \quad {D}(a) = \sum _v \widetilde{D}(v,a) \end{aligned}$$

where $I = \sum _i {\Pi }^i(1-{\Pi }^i)$, and ${\Pi }^i = \frac{\sum _v \kappa _v^{i}}{K^{t}}$ is the global *aspatial* share of population group *i* whilst $K^{i}$ and $K^{t}$ are the corresponding *aspatial* subgroup $K^{i}=\sum _v \kappa _v^{i}$ and total population $K^{t}=\sum _v \kappa _v^{t}$ of the entire study area.

*Exposure.*  Along the second *exposure dimension of segregation*, the two-group local $\widetilde{P}^{1,2}(v,a)$ and global ${P}^{1,2}$ exposure index is expressed according to

6 $$\begin{aligned} \widetilde{P}^{1,2}(v,a) = \frac{\kappa _v^{1}}{K^1}\widetilde{\tau }^2(v,a) \quad {\text{and}} \quad {P}^{1,2}(a) =\sum _v \widetilde{P}^{1,2}(v,a). \end{aligned}$$

In essence, the exposure index describes the potential interaction between population groups *i*^16^. Note that the index is normalized, i.e., its values span from 0 (minimum exposure) to 1 (maximum exposure). It must be emphasized that the original signal $\sum _i \widetilde{\tau }^i(v,a) \ne 1$ in Eq. (4), since $\widetilde{M}_{\kappa ^{t},a}(v)$ represents the total population, including all demographic groups between 0 and 80 and above (INSEE^34^). This choice is based on the fact that the correlation coefficients used in the bivariate multifractal analysis (Eqs. 10, 14) could otherwise (if the group proportions always summed to 1) only reproduce a perfect negative relationship. Although this argues against classical segregation approaches, wherein a two-group context $\sum _i \widetilde{\tau }^i(v,a)$ would add up to 1, $\widetilde{D}(v,a)$ and $\widetilde{P}^{1,2}(v,a)$, fortunately, provide almost identical results—with a Pearson correlation greater than 0.95—when calculated with the two types of approaches due to their robustness to grouping systems.

### Local multifractal analysis

The fundamental aspect of this approach involves applying traditional multifractal analysis to address geographical research questions and situations. This involves two key components: First, the transformation of data by a method that can effectively deal with spatial distributions that are *non-homogeneous* and is similar to a *wavelet* transform. At this first point, we also refer to earlier work in which the robustness of multifractal parameter estimations in the context of non-homogeneous point processes^36,37^ and the distinction between processes on the support and those on the mark^30^ are discussed in detail. The second step involves, the extraction of information about the behavior of *subsets of data within a local neighborhood* of arbitrary size. This feature has the potential to greatly increase the informativeness of the results for urban planning and policy-making and to improve comparability across different urban systems. The input for the multifractal analysis is then defined as the ratio of the population of group *i* and the total population at each origin point *v* ($\gamma _v^i$ in Eq. 7). Note that in segregation one is classically interested in the difference in the distribution of population proportions rather than the difference in the distribution of their absolute values since the latter is simply a function of the overall population density.

*Multiscale quantities.*  We begin by defining the multiscale quantities that play a crucial role in accurately estimating the fractal and multifractal parameters. Following the definition of the so-called sandbox coefficient originally developed by Tél et al.^38^ (also used since in numerous other applications such as in network science^39^), these quantities are centered on the original point process *v* to minimize edge effects. We first determine the wavelet-like transform $U^i(v,a)$ using the mean value $O^i(v,a)$ of the ratio $\gamma _v^i$ in a ball of radius *a* as

7 $$\begin{aligned} {\left\{ \begin{array}{ll} \gamma _v^i = \frac{\kappa _v^i}{\kappa _v^t}, \quad \quad \quad O^i(v,a) = \frac{1}{n_v(a)}\displaystyle \sum _{v', \; d_{v,v'}\le a }\gamma _{v'}^i, \quad \quad \quad O^i(v,\sqrt{2}a) = \frac{1}{n_v(a,\sqrt{2}a)}\displaystyle \sum _{v', \; a < d_{v,v'}\le \sqrt{2}a }\gamma _{v'}^i, \\ U^i(v,a) = O^i(v,a)-O^i(v,\sqrt{2}a). \end{array}\right. } \end{aligned}$$

where $n_v(a,\sqrt{2}a)$ is the number of observations *v* in a ring between the distances *a* and $\sqrt{2}a$. The use of the wavelet-like coefficients $U^i(v,a)$ for multifractal analysis is of critical importance because it introduces a new approach to segregation research that eliminates the importance of the local mean in the calculations and focuses instead on the dynamics of its local fluctuations; $\frac{1}{N_v}\sum _{v} U^i(v,a) = 0$. Mathematically, $U^i(v,a)$ (7) can be interpreted as a scalar product between the data (modeled by a weighted sum of Dirac masses) and wavelets, i.e. a well-localized continuous function with vanishing integral and of similar shape, centered at *v* and dilated to scale *a*. The use of such functions of similar shape that are shifted and dilated explains why the wavelet coefficients contain key information about the scaling invariance properties of the data (note, however, an important difference from the classical orthonormal wavelet basis approach, as elaborated in^21,32,33^, where wavelets have a constant shape, and are fitted to data defined on regular grids).

*Local scale-free analysis.*  While the classical or global scale-free analysis is based on the averaging of the multiscale quantities $U^i(v,a)$ over the entire available image, the *local* scale-free analysis^25,30^ is based on restricting averages within a window of radius $a' \equiv L$ (in Eqs. 1, 2, 3), centered on each original location $v(x_v, y_v)$. Geolocalization is thus performed using the distance decay function $w_{v,v'}(L)$ in Eq. (1). The selection of the parameter *L* is based on the idea that it must be larger than all the scales involved in the scale-free analysis, $a \le L$. The chosen value of *L* is selected in accordance with the largest scale of the range of scales in which the global scale-free analysis is observed. This is detailed at the beginning of the Results section. To ensure consistency between local environments, it is important to emphasize that the estimation for each original point (*v*) occurs within its own local environment. Consequently, no upscaling takes place during the application process. This means that the resolution of the original signal remains identical to that of the estimated multifractal parameters. As a result, each point reliably characterizes its own local segregation dynamics and maintains consistency as the analysis progresses from one point to the next. As in classical multifractal analysis, the first-order cumulant $C^i_1(v,a)$ is used to characterize the exponent that occurs most frequently in the data and represents the most frequently encountered level of local regularity $C^i_1(v,a)$:

8 $$\begin{aligned} C^i_1(v,a,L)= & \widetilde{M}_{\log |U^i|,L}(v,a),\,\, i = (1,2), \end{aligned}$$

9 $$\begin{aligned} C^i_1(v,a,L)&\thicksim _{a\rightarrow 0}&c^i_1(v,L)\log (a) + k^i_1(v),\,\, i = (1,2). \end{aligned}$$

In practice, $c^i_1(v,L)$ is estimated by performing linear regressions for each point $v(x_v, y_v)$ over a range of scales $a_{min} \le a \le a_{max} = L$. Univariate multiscale analysis can be extended to bivariate analysis, in which two signals $\kappa _{v}^i$ that are jointly defined or projected onto a common set of points are analyzed together. In bivariate scale-free analysis, the so-called wavelet coherence function $\rho _{ss}(v,a,L)$^40,41^, which is defined via the multiscale quanities $U^{1}(v,a)$ and $U^{2}(v,a)$, is derived as:

10 $$\begin{aligned} \rho _{ss}(v,a,L)=&\frac{\widetilde{M}_{U^{1}U^{2}, L}(v,a)- \widetilde{M}_{U^{1}, L}(v,a) \widetilde{M}_{U^{2}, L}(v,a)}{ \sqrt{ \widetilde{V}_{U^{1}, L}(v,a) \widetilde{V}_{U^{2}, L}(v,a) }}. \end{aligned}$$

$\rho _{ss}(v,a,L)$ enables the quantification of local scale-dependent cross-correlations between the spatial dynamics of the two processes.

*Multifractal analysis.*  In order to assess (cross-)dependencies that go beyond second-order (cross-)statistics (correlations), more elaborate multiscale quantities are also required, which are referred to as *p*-leaders and are defined in^42,43^; they allow us to derive important information on the size of the sets where the data analyzed have a given pointwise regularity exponent^42,43^. In the discrete and non-uniformly sampled case met in urban data, the corresponding definition is recast according to

11 $$\begin{aligned} Q^i(v,a,p)= & \left( \frac{1}{n_v(a)}\sum _{v', \; d_{v,v'}\le a }|U^i(v',a)|^p \right) ^{1/p}. \end{aligned}$$

The second-order cumulant $C^i_2(v,a)$ can then used to characterize multifractality. It makes it possible to estimate the width of the range, $c^i_2(v,L)$, of pointwise exponents present in data, as:

12 $$\begin{aligned} C^i_2(v,a,L)= & \widetilde{V}_{\log |Q^i|, L}(v,a),\,\, i = (1,2), \end{aligned}$$

13 $$\begin{aligned} C^i_2(v,a,L)&\thicksim _{a\rightarrow 0}&c^i_2(v,L)\log (a) + k^i_2(v),\,\, i = (1,2). \end{aligned}$$

In analogy to the wavelet coherence function $\rho _{ss}(v,a,L)$ defined above, a multifractal cross-correlation function $\rho _{mf}(v,a,L)$^21,32^ can also be defined locally, for each point $v(x_v,y_v)$, as:

14 $$\begin{aligned} \rho _{mf}(v,a,L)=&\frac{\widetilde{M}_{C_2^{1}C_2^{2}, L}(v,a)- \widetilde{M}_{C_2^{1}, L}(v,a) \widetilde{M}_{C_2^{2}, L}(v,a)}{ \sqrt{ \widetilde{V}_{C_2^{1}, L}(v,a) \widetilde{V}_{C_2^{2}, L}(v,a) }}. \end{aligned}$$

$\rho _{mf}(v,a,L)$ permits to quantify local scale-dependent cross statistical dependencies not already encoded by local scale-dependent second order statistics and cross correlations $\rho _{ss}(v,a,L)$. Therefore, $\rho _{mf}(v,a,L)$ provides independent, complementary and enriched information on local scale-free dynamics, compared to $\rho _{ss}(v,a,L)$. Finally, global scalings for all the above-defined fractal and multifractal parameters simply stem from the average over all points *v* as defined in the supporting information in Eqs. (S16).

### Critical differences between the classical and multifractal indices

The similarities and differences between the two methods of classical segregation (CS) and multifractal (MF) analysis are numerous, but for better interpretability of the results, it is important to highlight some of them here.

1. *Point of geolocalization. * CS measures use the distance decay function $w_{v,v'}(a)$, i.e. geolocalization according to Eq. (1), to derive their multiscale quantities $\widetilde{\tau }^i(v,a)$ (Eq. 4) but the calculation of the indices $\widetilde{D}(v,a)$ (Eq. 5) and $\widetilde{P}^{1,2}(v,a)$ (Eq. 6) otherwise remains at the pixel level. Geolocalization with distance decay function in MF analysis is first performed with the introduction of the local neighborhood *L*, i.e. using $w_{v,v'}(L)$ in Eq. (1), which is the resolution at which all fractal and multifractal properties are computed (Eqs. 8, 12, 10, 14).

2. *Multiscale quantity. * Second, it must be emphasized that the main *similarity* between the two methods is that they both consider the local distribution of population densities — $\widetilde{M}_{\kappa ^{i}, a}(v)$, $\widetilde{M}_{\kappa ^{t}, a}(v)$ and $O^i(v,a)$ — as the main building blocks for the analysis. However, the CS index $\widetilde{P}^{1,2}(v,a)$ only uses a low-pass filter $\widetilde{\tau }^i(v,a)$ (Eq. 4), or the ratio of the localized spatial averages, for its calculation. In the case of the CS measure $\widetilde{D}(v,a)$ (Eq. 5), this is further adjusted as the difference between the latter $\widetilde{\tau }^i(v,a)$ (Eq. 4) and the global average $\Pi ^i$ according to $\widetilde{\tau }^i(v,a) - \Pi ^i$. Therefore, as with most smoothing methods, it remains essentially a low-pass-like filter as well; as the scale *a* increases, the dissimilarity between the values decreases due to the averaging of nearby values. This is true even after the removal of the constant $\Pi ^i$. MF analysis goes one step further and considers the local variations of average population intensities $O^i(v,a)$, i.e., it uses the bandpass wavelet technique $U^i(v,a)$ (Eq. 7). The next section will show how this difference between low-pass, “low-pass-like” and band-pass filters can lead to fundamentally different results.

3. *Definition of locality. * Another important difference arises from their intuition to detect spatial heterogeneities. Classical segregation works with the aforementioned comparison between the local and global levels, thus it is sensitive to MAUP issues. Multifractal analysis summarizes results for arbitrary local spatial environments *L*, i.e., it is completely tract-free.

4.*Range of values. * Finally, both techniques employ approaches to scale indices between 0 and 1 (CS) or between $-1$ and 1 (MF) to ensure comparability between case studies. Essentially, both CS indices discussed here use the ratio of local (in our case pixel-level) to global (metropolitan-level) population for normalization, which makes them susceptible to pixel-level population density, as explained in detail in the results section. In contrast, the MF parameters do not require any additional normalization techniques as they are correlation coefficients.

To summarize, the first critical difference in the calculation lies in the derivation of the multiresolution quantities, with the CS measures using a more low-pass-type filter and the MF measures using a band-pass filter. This has a strong impact on the ability to motivate progression across scales. The second concerns the difference between a local-global comparison method (CS) and a strictly local cross-correlation approach (MF) to segregation, which affects stability with respect to MAUP issues and local population densities.

**Fig. 2:**
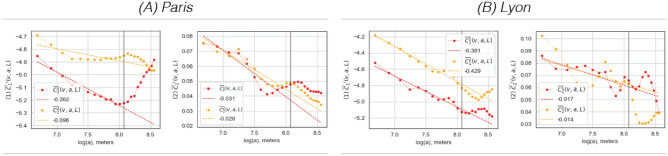
*Global scaling until 5000 m;* for the univariate multifractal parameters $\overline{C}_1^i(v,a,L)$ (1) and $\overline{C}_2^i(v,a,L)$ (2) (Eqs. S.16) for the two signals; the share of population under 18 ($i=1$) and the share of people over 65 ($i=2$). There is a clear regime change in the scaling for $\overline{C}_1^i(v,a,L)$ (in Paris, **A**) and $\overline{C}_2^i(v,a,L)$ (in Lyon, **B**) at about $a=3200$ m, marked with a gray vertical line. This scale of $a=3200$ is therefore chosen as the largest scale for fitting the linear regression models.

## Results

With data defined on a $200 \times 200$ meter grid, global scale-free dynamics exists on a range scale *a* spanning from $a_{min} = 800$ to $a_{max} = 3200$ m. The corresponding local environment for the multifractal analysis are thus set to $L=3200$, the maximum value of the scales *a* to keep the analysis as “local” as the selected scale range allows. The size of the local environment is indicated in Fig. 1 with black dashed circles. The largest scale of $a_{max} = 3200$ m was chosen according to the global scaling of the univariate multifractal parameters in Fig. 2 since a clear change in the scaling regime can be observed here (gray vertical line). Let us begin by examining the demographic characteristics depicted in Fig. 1, focusing on two datasets: the share of under 18 and over 65-year-olds. For the three scales presented — the lowest, the mid-range, and largest observed scales — for the non-weighted local average $O^i(v,a)$, it appears that the southern regions of the municipality of Paris in Fig. 1A, have a significant concentration of persons aged 65 and over, while the outer districts have a higher prevalence of families with children. In our second case study, Fig. 1B shows that Lyon exhibits similar dynamics to Paris in its suburbs, i.e. there is a clear offset in the prevalence of the two population groups. This is particularly pronounced in the east and south-east, where more families with children seem to have settled, while the over-65s are under-represented. In contrast to the Paris region, however, both groups are quite uncommon in the city center of Lyon. This important difference between the two signal pairs is also reflected in Table 1, where the global correlation coefficient for Paris between under 18-year-olds and over 65-year-olds is $-0.81$, while it is significantly weaker for Lyon at $-0.54$.

**Fig. 3:**
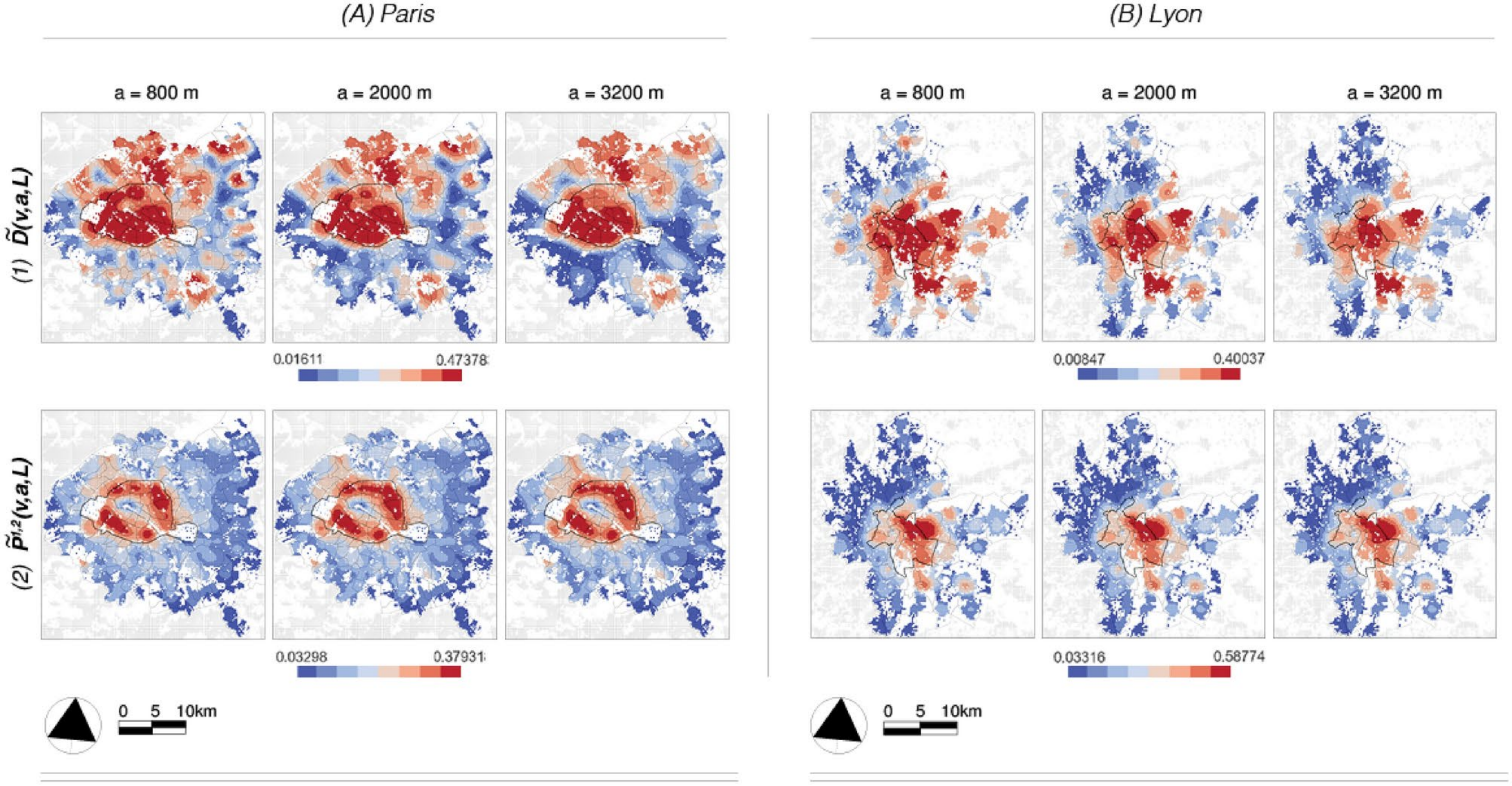
*Classical segregation* results for the dissimilarity index $\widetilde{D}(v,a,L)$ (1) and the exposure index $\widetilde{P}^{1,2}(v,a,L)$ (3) at three length scales *a*; the finest, the middle, and the largest observed scales. In contrast to the results of the bivariate multifractal analysis, the patterns that arise on the different scales are fairly similar. While segregation according to $\widetilde{D}(v,a,L)$ is concentrated within the city limits of Paris (A) and in the northern suburbs, the interactions $\widetilde{P}^{1,2}(v,a,L)$ are most pronounced in a ring-shaped structure along the city boundary. In Lyon (B), $\widetilde{D}(v,a,L)$ values within the municipality are also elevated. Exposure $\widetilde{P}^{1,2}(v,a,L)$ is highest in the eastern parts of the city and extends mainly to the eastern and southern suburbs.

### Classical segregation indices

The classical segregation measures used here (see Eqs. 5, 6) ultimately measure the contribution of each locality to the global configuration. Accordingly, the localized results at the points *v* are of very low magnitude and difficult to interpret on the map legends; for illustrative purposes, we, therefore, multiply each value $\widetilde{D}(v,a)$ and $\widetilde{P}^{1,2}(v,a)$ in Fig. 3 by the constant $N_v$. Note also that the classical measurements work at the $200\times 200$ m cell level, while the multifractal results are at the *L* resolution level. Therefore, we also apply spatial smoothing with a sliding window of radius $a'= L$ (in Eq. 1 and Eq. 2) according to $\widetilde{D}(v,a,L)= \widetilde{M}_{\widetilde{D}, L}(v,a)$ and $\widetilde{P}^{1,2}(v,a,L)= \widetilde{M}_{\widetilde{P}^{1,2}, L}(v,a)$ for better visual comparability of the two methods. The original pixel-level results for $\widetilde{D}(v,a)$ and $\widetilde{P}^{1,2}(v,a)$ can be found in the supporting material in Figure S3.

**Fig. 4:**
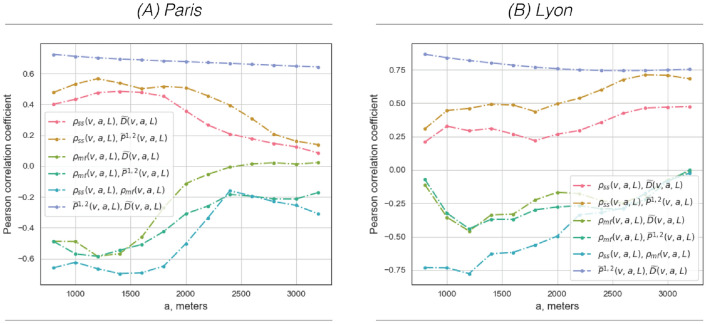
*Correlation between the results of classical segregation and multifractal analysis;* the strength of the correlation decreases significantly towards larger scales, with the exception of the correlation between $\rho _{ss}(v,a,L)$ and the classical segregation results, which increases slightly. The relationship between $\widetilde{D}(v,a,L)$ and $\widetilde{P}^{1,2}(v,a,L)$ is strongly positive and stays persistent.

*General observations. * Figure 3 shows the results obtained for the chosen classical segregation measures. Before we discuss the case studies in detail, let us begin with two important and more general observations. (**1**) Locally, the progression of both the $\widetilde{D}(v,a,L)$ and $\widetilde{P}^{1,2}(v,a,L)$ index across length scales *a* is notably insignificant. This observation is also supported by the general lack of scale-dependent progression in Figure S4 in the supplementary material, which shows the local scaling for the CS parameters at each location. In other words, local segregation results are not really local: they are hardly dependent on the scale *a* as also reflected in the fact that their Pearson correlation coefficient across scales remains constant in Fig. 4 (dark blue curves). This result is due to the choice of their “low-pass” multiscale quantity, which allows little multiscale progression and essentially consists of incrementally “smoothing out” local differences. (**2**) The revealed patterns on visual inspection show a remarkable similarity to the local distribution of population densities shown in Figure S2. This applies in particular to $\widetilde{P}^{1,2}(v,a,L)$, but can also be observed in the inner city contexts for $\widetilde{D}(v,a,L)$. This therefore indicates a strong possible bias in the CS indices in relation to local population density.

*Case studies. * Figure 3.1 (top) shows the results of the spatialized $\widetilde{D}(v,a,L)$ index. The municipality of Paris and its northern suburbs and the municipality of Lyon and its eastern and southern suburbs stand out as a dark red area with the highest values; High $\widetilde{D}(v,a,L)$ values indicate a significant contribution to the global index (see also *D*(*a*) in Eq. 5) in which one or the other population group is overrepresented compared to the average distribution at the system level and is, therefore, the most segregated. The exposure index $\widetilde{P}^{1,2}(v,a,L)$ in Fig. 3.2 (bottom) indicates the potential interaction between population groups; in this case, the darker the red color, the greater the exposure of under 18 year-olds ($i=1$) to those over 65 ($i=2$) year-olds. In Fig. 3.2.A (in the Paris region) a notable ring, which largely coincides with the municipal boundary, is clearly visible, i.e., this is where the two subpopulation groups are most likely to be in contact with each other. In Fig. 3.2.B (in Lyon), most interactions take place in the eastern part of the city, spilling over into the neighboring suburbs. Note that this index is asymmetric, i.e. $\widetilde{P}^{2,1}(v,a,L)$ may reveal different features, but we omit them here as they strongly resemble $\widetilde{P}^{1,2}(v,a,L)$ discussed earlier. On visual inspection, Figs. 3.1 and 3.2 seem to convey opposing tendencies within both the city of Paris and Lyon, while they largely coincide in their outer districts. However, we emphasize that the validity of this finding remains unclear when unevenness/interaction coincides locally with high population density (see the discussion on normalization in the methodology section). Nevertheless, it should be underlined that the two dimensions of segregation, evenness and exposure, are empirically closely related but conceptually distinct, since the former is based on the relative “size” of the groups being compared, whereas the latter is not ^44^.

**Fig. 5:**
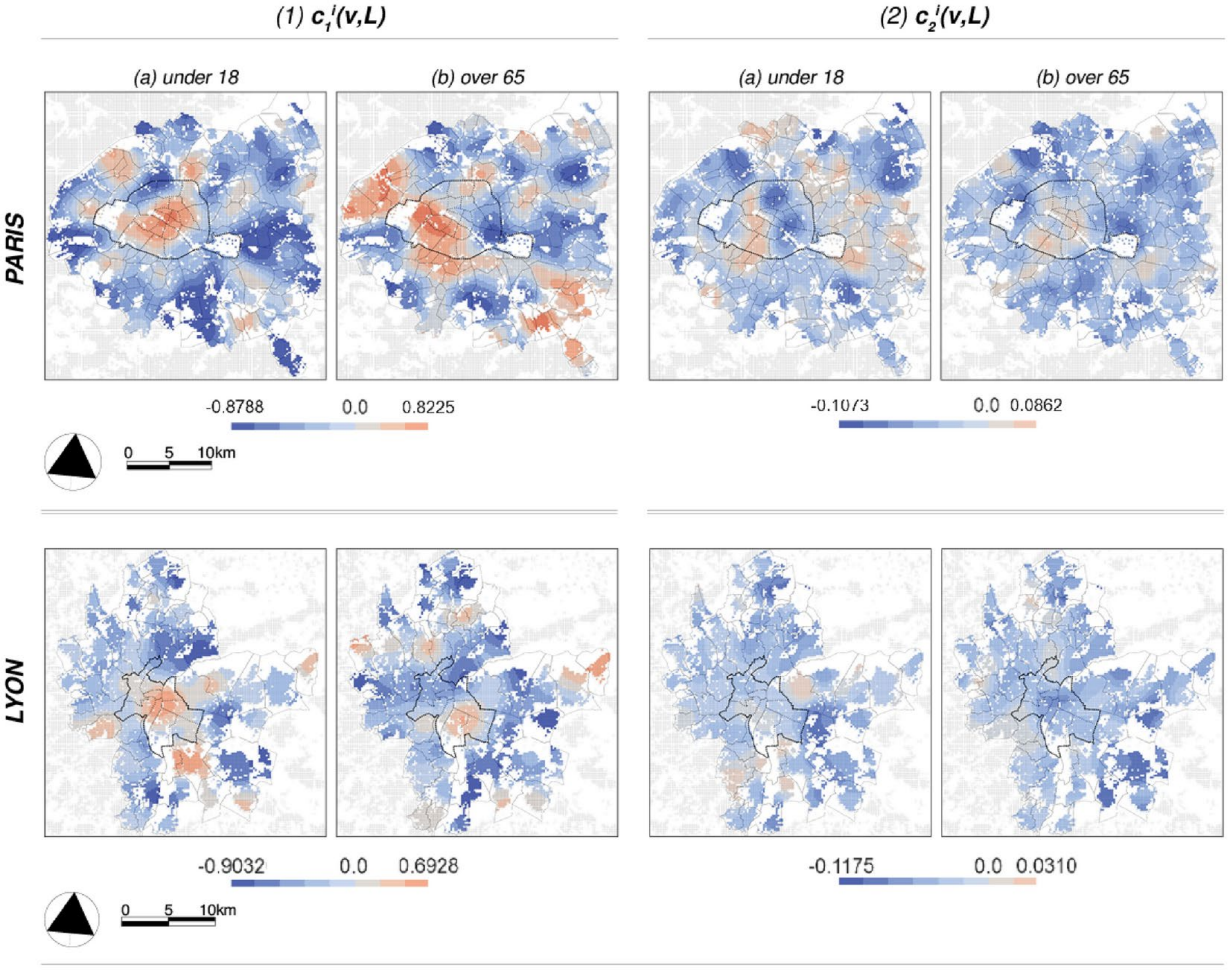
*Univariate multifractal analysis results*: the local estimations for $c_1^i(v,L)$ (1) and $c_2^i(v,L)$ (2) obtained using all available scales between 800 and 3200 m for the case studies of Paris (top) and Lyon (bottom). (**a**) shows the results for the share of people under 18 ($i=1$), and (**b**) shows the results for the share of people over 65 ($i=2$).

### Univariate multifractal analysis

In the univariate context, the critical parameters to be discussed are $c_1(v,L)$ and $c_2(v,L)$, while in the bivariate context their respective correlations $\rho _{ss}(v,a,L)$ and $\rho _{mf}(v,a,L)$ are of central importance. Because the focus of this article is on the bivariate setting, the univariate MF results are presented only briefly. Figure 5.1 (for Paris and Lyon, respectively) shows the estimates for the order one $c_1(v,L)$ cumulants, which characterizes the development of $C^i_1(v,a,L)$ across scales, or long-range dependencies in the two signals using all available scales between 800 and 3200 m; higher values mean a higher smoothness, or more easily recognizable large-scale spatial trends in population distribution, and hence stronger dependencies over successive length scales *a*. For both case studies, the signal is generally smoother within the municipal boundary and becomes rougher and rougher (or the connections are more short-distance concentrated) in a rather concentric manner for the under-18s. In contrast, the signal for the over-65s shows a south-north gradient within the city of Paris and an east-west gradient within Lyon. Regarding the global average scaling behavior for the entire study areas combined, in Fig. 2.B we observe generally steeper negative slopes in Lyon, meaning that dependencies remain strong over shorter distances than in the Paris region. This can also be seen in Figure S5, where the maximum of the theoretical spectra of Paris (black curves) lies exactly at the derived values in Fig. 2.A.1 and to the right of those of Lyon, which peak at the values in Fig. 2.B.1.

Figure 5.2 displays the $c_2(v,L)$ estimates using the same scale range of 200 to 3200 m; the darker the blue color, the more intermittent the signal in the local neighborhood of size *L*. Essentially, $c_2(v,L)$ describes how the probability of the occurrence of extreme local fluctuations (in the regularity of the data) increases with the development of the scales. If $c_2(v,L) < 0$ (blue colors), the probability of extreme events increases when *scales decrease*. In other words: $c_2(v,L)$ captures *local* deviations from the corresponding value of the *most commonly occurring* regularity $c_1(v,L)$ or the degree of intermittency around this value. Starting with the under-18s, the highest intermittency in Paris is found in the eastern parts of the city. In Lyon, it is the suburbs that show some areas of high intermittency, particularly in the east and north. Lastly, the signal for the over-65s in the municipality of Paris shows no multifractality, while it is highly intermittent in the city of Lyon. As for the global behavior in the overall case studies, Fig. 2.A.2 and B.2 shows that both processes are slightly more intermittent in the Paris region. This can also be seen in Fig. S5, where the spectra (black curves) of Paris show a greater width, i.e. a higher degree of multifractality, than the spectra of Lyon in orange. To summarize, both $c_1(v,L)$ and $c_2(v,L)$ reveal certain important structural properties of the observed phenomena as a function of spatial scale.

**Fig. 6:**
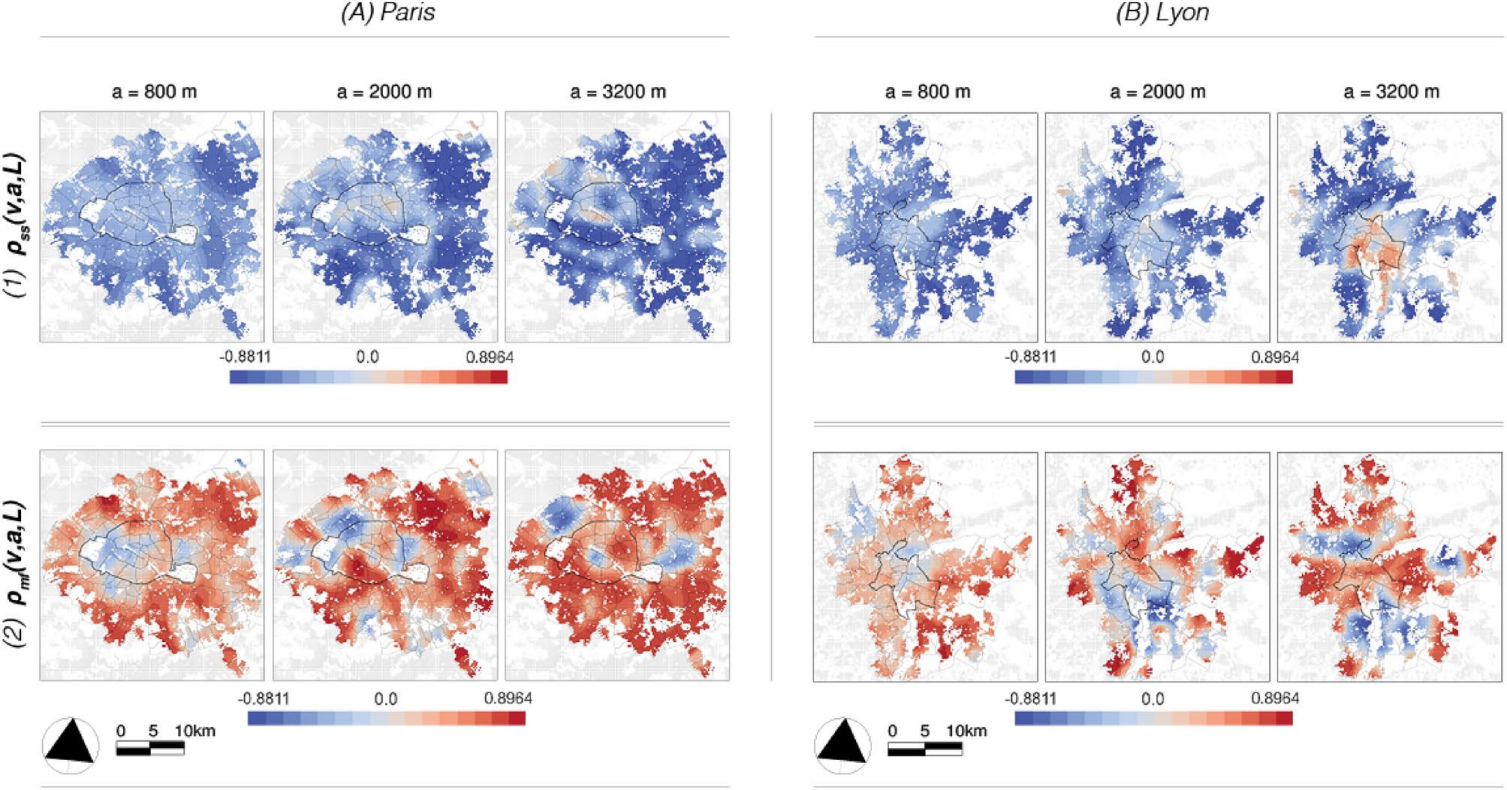
*Multifractal cross-correlation results* for $\rho _{ss}(v,a,L)$ (1) and $\rho _{mf}(v,a,L)$ (2) at three length scales *a*; the finest, the middle, and the largest observed scales. While $\rho _{ss}(v,a,L)$ takes primarily negative values, the values of $\rho _{mf}(v,a,L)$ vary between minus 1 and 1. In contrast to the results of classical segregation, the patterns that emerge on the different scales are rather different.

### Results of the bivariate multifractal analysis and their comparison with classical segregation measures

Figure 6 contains the results for the bivariate multifractal parameters. Following the discussion of the CS results, we first address four more general observations before discussing the case studies in more detail.

*General observations.  * (**1**) If we start with the MF results in Fig. 6, it is noticeable at first glance that $\rho _{ss}(v,a,L)$ takes predominantly negative values, while $\rho _{mf}(v,a,L)$ fluctuates between $-1$ and 1, i.e. between negative and positive correlation. (**2**) Figure 4.A for Paris shows that the two types of analysis—classic segregation and multifractal analysis—are generally most strongly correlated between 800 and 2000 m. This is reciprocated in metropolitan Lyon for the relationship between $\rho _{mf}(v,a,L)$ and the two CS indices, but the opposite can be observed for $\rho _{ss}(v,a,L)$, with the correlation rising with increasing scale *a*. The latter is due to a slightly different dynamic in this region, where $\rho _{ss}(v,a,L)$ takes on positive values at large scales (Fig. 6), which will be further discussed below. (**3**) The evolution of the MF results over the scales *a* is rather dynamic, which will be reflected in the different appearance of the maps in Fig. 6 as well as on the local and global scaling in Figure S4.2. Moreover, we observe different dynamics not only through the latter scales, but also from one spatial location *v* to another. The latter two points together constitute an important finding of the present work, as they indicate that the socio-spatial data collected at the metropolitan level cannot be considered as a homogeneous multifractal process. It is important to point out here that, as shown in previous works^21,32,33,36,41^, $\rho _{ss}(v,a,L)$ and $\rho _{mf}(v,a,L)$ would be approximately constant functions along the scales *a* if they were applied to, for example, synthetic multifractal stochastic processes with locally homogeneous structural properties. (**4**) Finally, and in contrast to the CS results, the revealed patterns in the MF context in Fig. 6 bear hardly any resemblance to the population density shown in Figure S2.

*Case studies. * In general, we now distinguish between the three scales shown, each of which roughly includes three types of areas; *[T.1]* Decorrelation both in $\rho _{ss}(v,a,L)$ and $\rho _{mf}(v,a,L)$, *[T.2]* Strong negative correlation in $\rho _{ss}(v,a,L)\lesssim -0.75$ and strong positive correlation in $\rho _{mf}(v,a,L)\gtrsim 0.75$, *[T.3]* Decorrelation in $\rho _{ss}(v,a,L) \sim 0$ and negative correlation in $\rho _{mf}(v,a,L) < 0$.

*Neighbourhood-level dynamics (a = 800 m). * Staying at the finest scales and focusing on the municipalities of Paris and Lyon, we find that both $\rho _{ss}(v,a,L)$ and $\rho _{mf}(v,a,L)$ can be characterized by decorrelation or values close to 0 (Fig. 6), i.e, areas of type *[T.1]*. At the same time, these are areas that typically correspond to higher exposure values $\widetilde{P}^{1,2}(v,a,L)$ in relation to all scales *a*. The latter suggests that decorrelation in the multifractal context signals certain unstructured mixing processes in which the subgroups may be “overexposed” in a locally diverse manner. Therefore, decorrelation in spatial dynamics may be directly linked to more potential interaction among population segments.

*District-level dynamics (a = 2000 m). * In Fig. 6 at 2000 m, we begin with the results of the self-similar cross-correlation function $\rho _{ss}(v,a,L)$. The territorial extent of decorrelation *[T.1]* in both case studies decreases. In Paris, it is now observed mainly in the northern half of the city and to its northwest. In contrast, a strong negative correlation *[T.2]* monopolizes the eastern and southwestern outskirts and the southern municipality. In Lyon, decorrelation *[T.1]* dominates the municipality as well as certain distinct radial segments that branch out in every direction. Areas of stronger negative correlation *[T.2]* are embedded between these segments. Generally, a strong negative correlation in $\rho _{ss}(v,a,L)$ signals non-uniformity, but in the *dynamics* of the two processes, i.e. where one population group shows growth in space, the other decreases. In both the Lyon region and the Paris region, these areas with negative correlation in $\rho _{ss}(v,a,L)$ or the dark blue colors in Fig. 6.1 (top) largely correspond to a strong positive correlation *[T.2]* in $\rho _{mf}(v,a,L)$ or the dark red colors in Fig. 6.2 (bottom), indicating that both signals are also locally and jointly intermittent. Further, in Fig. 6.2, there are particular regions of the $\rho _{mf}(v,a,L)$ results—at all scales—that have negative values *[T.3]* or blue colors. These may indicate certain interesting dynamics, where one signal is intermittent or has some important local bursts or concentrations, while the other signal is more regularly distributed over the area; $\rho _{mf}(v,a,L)<0$ may thus be an indication of *intermittent segregation processes*. Since this interpretation is difficult to prove using existing geographic analysis methods, we consider *[T.3]* areas in great detail in the next subsection.

*City-level dynamics (a = 3200 m). * Finally, concerning the largest observed scale $a=3200$ in Fig. 6, we observe a further decrease in decorrelation for both parameters, while strongly negative $\rho _{ss}(v,a,L)$ and positive $\rho _{mf}(v,a,L)$ dominate *[T.2]*. This is because the two signal dynamics are globally negatively correlated and jointly intermittent. Thus, as we progress to larger and larger scales, this behavior will be the most influential over the territory. An important exception is the southern region of Lyon, which is largely characterized by positive $\rho _{ss}(v,a,L)> 0$ (red color) on this scale, indicating that both population groups exhibit spatial growth.

**Fig. 7:**
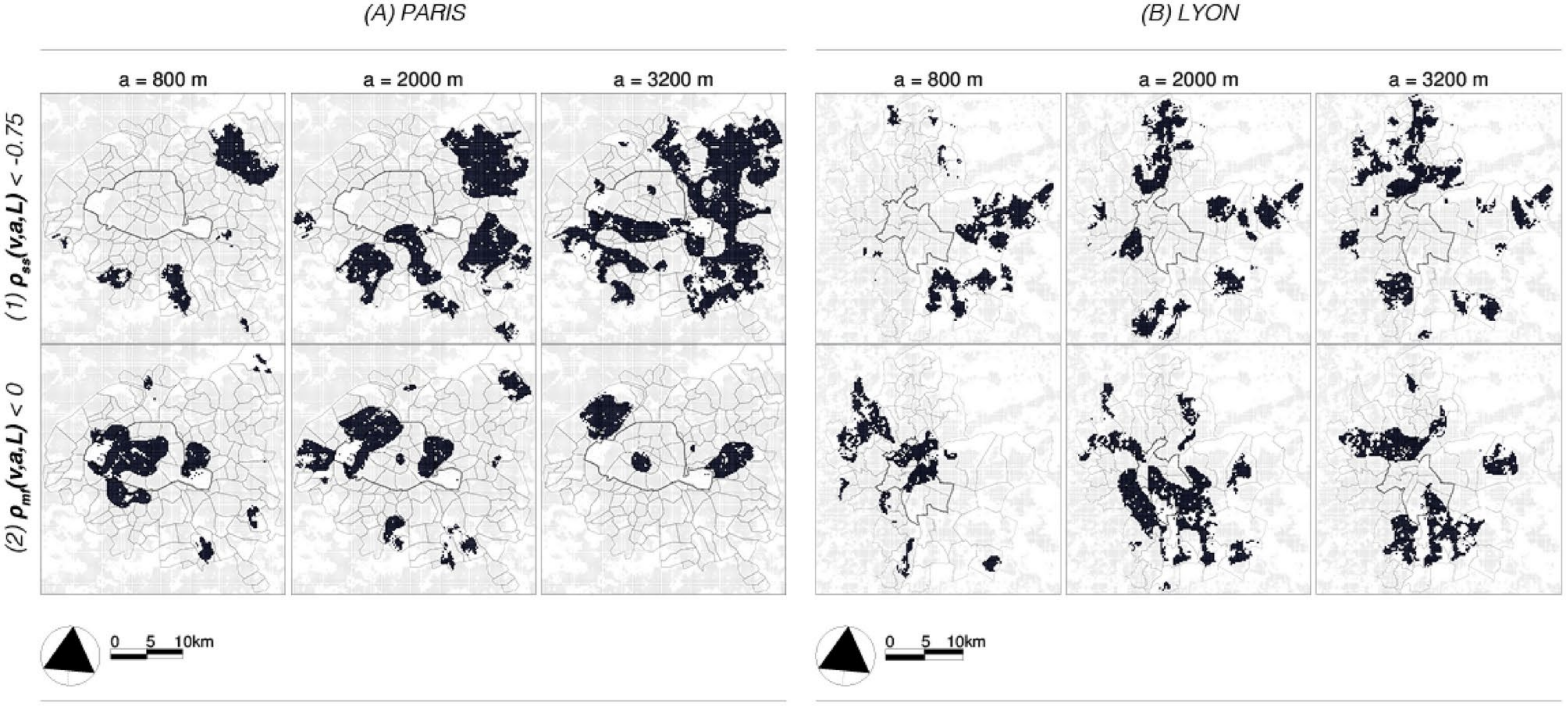
*Selected value ranges from the bivariate multifractal analysis results*. (1) Strong negative correlations in $\rho _{ss}(v,a,L)$ or in the top row can be associated, e.g., with the classical unevenness results. In contrast to the $\widetilde{D}(v, a)$ index, the results are not disturbed by local population intensities. (2) Negative $\rho _{mf}(v,a,L)$ results reveal a new axis for segregation information that is not accessible with classical measures. These are regions that are predominantly dominated by one population group, but where important localized “outbreaks” or concentrations of members of the other group may occur.

### Unevenness, exposure and “intermittent” segregation

To further facilitate the interpretation of the multifractal results, we only consider certain specific value ranges in Fig. 7. The highlighted (black) areas in Fig. 7.1 (top) largely correspond to the *[T.2]* typologies and the highlighted areas in Fig. 7.2 (bottom) to the *[T.3]* typologies from the previous subsection. First, in the suburban regions, Fig. 7.1 shows agreement with the results of dissimilarity of both case studies in Fig. 3.1, i.e. where $\rho _{ss}(v,a,L)<-0.75$, dissimilarity $\widetilde{D}(v,a,L)$ and thus segregation is elevated. Note that these areas also show low exposure in Fig. 3.2, suggesting a low level of local interaction. The self-similar cross-correlation $\rho _{ss}(v,a,L)$ thus suggests that opposing trends in spatial dynamics may be directly associated with higher and more pronounced segregation processes, both in terms of fewer possible interactions (i.e., exposure) and higher dissimilarity (i.e., evenness). There are however also notable differences between strongly negative $\rho _{ss}(v,a,L)$ and high values of $\widetilde{D}(v,a,L)$ in the densely populated areas, but these may be due to the normalization in the CS context and could therefore be considered $\widetilde{D}(v,a,L)$ artifacts. However, the discussion of this effect is beyond the scope of this article. In Fig. 7.2 we have highlighted areas where $\rho _{mf}(v,a,L)<0$ *[T.3]*, i.e. where “intermittent” segregation may be taking place;

*Neighbourhood-level dynamics, (a = 800 m). * At $a=800$ in Paris, the intermittent segregation is higher in areas with middle/higher incomes and an older population (see also Figure S1). At the same time, these are also neighborhoods known to be popular with students, such as the so-called “Latin Quarter”. In our second case study of Lyon, we see, among others, that the traditionally working class but now highly gentrifying “Croix-Rousse” is highlighted. These two examples cannot be considered segregated as a whole in the classical sense, but the mixture of populations is not homogeneous. We thus see that the MF cross-correlation parameter $\rho _{mf}(v,a,L)<0$ can signal certain important and locally well-known particularities in the dynamics. It is important to emphasize that such features of segregation have so far been difficult to uncover with CS measures.

*District-level dynamics, (a = 2000 m). * Starting again with Paris at the *district* level $a=2000$, there are two notable regions, where the area with $\rho _{mf}(v,a,L) < 0$ increases significantly in comparison to $a=800$ (Fig. 7.2). The first of these areas corresponds to the northwestern districts and inner suburbs. In contrast to the declining population figures within the city of Paris, these dense areas of the Hauts-de-Seine department are experiencing steady population growth thanks to a relatively high birth rate and the influx of many young households (see also Figure S1) attracted by concentrated top-down urban planning activities. The second area is located east of Paris and includes the municipalities of Montreuil and Bagnolet in the Seine-Saint-Denis department. According to INSEE^34^, these former working-class neighborhoods have experienced some of the strongest population growth in the entire Ile-de-France region at the beginning of the 2010s. They are also known as recent examples of gentrification areas^45,46^ (although the process started in the 2000s), marked by the arrival of highly educated people, rising real estate prices, and several urban renewal projects. Similar tendencies can be observed in Lyon; Ambitious urban redevelopment projects such as the Lyon Confluence area are being accentuated by $\rho _{mf}(v,a,L) < 0$.

*City-level dynamics, (a = 3200 m). * Finally, in the largest “city” scale, $a=3200$, the black areas (in Fig. 7.2) mark the centers of a completely different global dynamic around them, in which the two signals are characterized by opposing intermittency trends. In the Paris region, this is the case for Fontenay-sous-Bois, Nogent-sur-Marne, and Le-Perreux in the east, as well as Levallois-Perret, Asnière-sur-Seine, Courbevoie, and La Garenne-Colombes, which attract many middle and upper-class households with children looking for larger apartments and possibly single-family homes with gardens close to Paris. Like many towns around Paris, these towns also have an aging population; So, on this scale, these cities have localized concentrations of younger households with children amidst an aging population. The same constellation can be observed in the Lyon region, even in districts or cities where the average income is slightly higher than in the rest of the conurbation. In the north, the golden suburbs such as Saint-Didier-au-Mont-d’Or, Champagne-au-Mon-d’Or, and Dardilly stand out: they are aging towns that are increasingly attracting households with children. In summary, the underlying social and economic processes in these areas may be very different, but in each of the cases, they indicate extraordinary spatial dynamics that may require immediate attention from urban policy and planning. To summarize, the relationship between the two methods can therefore be derived as follows;

1. *[T.1]* $\rho _{ss}(v,a,L)\sim 0$ & $\rho _{mf}(v,a,L)\sim 0$. High levels of interaction and local mixing, correspondence to high $\widetilde{P}^{1,2}(v,a,L)$.
2. *[T.2]* $\rho _{ss}(v,a,L) \lesssim -0.75$ & $\rho _{mf}(v,a,L) \gtrsim 0.75$.“Classical” segregation (Fig. 7.1), i.e., high $\widetilde{D}(v,a,L)$ and low $\widetilde{P}^{1,2}(v,a,L)$.
3. *[T.3]* $\rho _{ss}(v,a,L)\sim 0$ & $\rho _{mf}(v,a,L)< 0$. “Intermittent” segregation (Fig. 7.2), inaccessible with CS measures.

## Discussion

This study investigated whether an intrinsic multiscale framework in urban analytics, i.e., bivariate multifractal analysis, can provide some new insights in the context of two-group segregation research which has so far only been able to independently observe the behavior of certain selected parameters from scale after scale. Our first result showed a characteristic scale in the univariate processes themselves, which ultimately defined the range of the analysis and the size of the local environment for the local analysis. Second, we saw that, indeed, bivariate multifractal results share some common features with classical segregation results; on fine scales, measures could be associated with the exposure index $\widetilde{P}^{1,2}(v,a)$ and the chosen evenness index $\widetilde{D}(v,a)$. At the same time, both of the bivariate multifractal parameters showed remarkable progression across the observed scales, i.e., the patterns revealed were highly dissimilar due in part to the use of the wavelet technique. For example, the southern districts of Paris showed marked mixing and increased interaction at $a=800$ m but soon switched to relatively high segregation at about $a=2000$. Such results could provide important information *for the appropriate scale* of urban policy and planning action. It is important to emphasize that progression across scales was largely absent in the classical multilevel segregation descriptions. Moreover, contrasting spatial dynamics revealed by $\rho _{ss}(v,a,L)$ could be related to stronger segregation, while the dynamic features of $\rho _{mf}(v,a,L)$ added important new information to the classical segregation results. They revealed areas of unusual dynamics, possibly with different underlying causes, where socioeconomic processes with very different levels of intermittency coincide. Using a detailed analysis of three spatial snapshots, we determined that spatial infiltration or exfiltration processes may occur in these “intermittently” segregated regions. In general, the fact that the bivariate multifractal results differed from scale to scale and from one spatial location to another underlines that urban systems cannot be treated as homogeneous multifractal processes—which is also clearly confirmed by the univariate multifractal results—and therefore a local analysis approach is indispensable. Finally, unlike many existing local analysis approaches, the methodology presented here was completely independent of the delimitation of the global case study, so that cross-comparisons between different sites and different urban subsystems can be carried out in a highly transparent manner.

To introduce the methodology, the Paris and Lyon metropolitan area was chosen as case studies in this article. However, tests with other datasets and additional urban subsystems, possibly from entirely different national contexts, are essential next steps. It should also be noted that the multiscale variables assume that urban areas are rotationally invariant. Therefore, the development of other related mathematical and geographic concepts that can more realistically account for the inhomogeneity of the urban environment is urgently needed.

## Supplementary Information

Supplementary Information.

## Data Availability

The data are publicly available on the website of the French census database INSEE: https://www.insee.fr/fr/statistiques/4176290?sommaire=4176305.

## References

[1] Reardon, S. F. & Oâ’Sullivan, D. Measures of spatial segregation. *Sociol. Methodol.* **34**, 121–162 (2004).

[2] Morrill, R. L. On the measure of geographic segregation. *Geography Res. Forum* **11**, 25–36 (1991).

[3] Stepinski, T. F. & Dmowska, A. Complexity in patterns of racial segregation. *Chaos Solitons Fractals* **140**, 110207 (2020).

[4] Reardon, S. F. & Firebaugh, G. Measures of multigroup segregation. *Sociol. Methodol.* **32**, 33–67 (2002).

[5] Massey, D. S. & Denton, N. A. The dimensions of residential segregation. *Soc. Forces* **67**, 281–315 (1988).

[6] White, M. J. The measurement of spatial segregation. *Am. J. Sociol.* **88**, 1008–1018 (1983).

[7] Wong, D. W. Spatial indices of segregation. *Urban Stud.* **30**, 559–572 (1993).

[8] Logan, J. R., Stults, B. J. & Farley, R. Segregation of minorities in the metropolis: Two decades of change. *Demography* **41**, 1–22 (2004). 15074122 10.1353/dem.2004.0007

[9] Chae, J. 7-day patterns in black-white segregation in 49 metropolitan areas. *Sci. Rep.* **14**, 6740 (2024). 38509129 10.1038/s41598-024-56257-1 PMC10954647

[10] Sakoda, J. M. A generalized index of dissimilarity. *Demography* **18**, 245–250 (1981). 7227588

[11] White, M. J. Segregation and diversity measures in population distribution. *Popul. Index* **6**, 198–221 (1986). 12340704

[12] Wong, D. W. Spatial decomposition of segregation indices: A framework toward measuring segregation at multiple levels. *Geogr. Anal.* **35**, 179–194 (2003).

[13] Reardon, S. F. et al. The geographic scale of metropolitan racial segregation. *Demography* **45**, 489–514 (2008). 18939658 10.1353/dem.0.0019 PMC2831394

[14] Neira, M., Molinero, C., Marshall, S. & Arcaute, E. Urban segregation on multilayered transport networks: A random walk approach. *Sci. Rep.* **14**, 8370 (2024). 38600261 10.1038/s41598-024-58932-9 PMC11006669

[15] Barros, J. & Feitosa, F. F. Uneven geographies: Exploring the sensitivity of spatial indices of residential segregation. *Environ. Plan. B Urban Analyt. City Sci.* **45**, 1073–1089 (2018).

[16] Feitosa, F. F., Camara, G., Monteiro, A. M. V., Koschitzki, T. & Silva, M. P. Global and local spatial indices of urban segregation. *Int. J. Geogr. Inf. Sci.* **21**, 299–323 (2007).

[17] Petrović, A., van Ham, M. & Manley, D. Multiscale measures of population: Within-and between-city variation in exposure to the sociospatial context. *Ann. Am. Assoc. Geogr.* **108**, 1057–1074 (2018).

[18] Ardestani, B. M., O’Sullivan, D. & Davis, P. A multi-scaled agent-based model of residential segregation applied to a real metropolitan area. *Comput. Environ. Urban Syst.* **69**, 1–16 (2018).

[19] Sousa, S. & Nicosia, V. Quantifying ethnic segregation in cities through random walks. *Nat. Commun.* **13**, 5809 (2022). 36192428 10.1038/s41467-022-33344-3 PMC9530170

[20] Lee, B. A. et al. Beyond the census tract: Patterns and determinants of racial segregation at multiple geographic scales. *Am. Sociol. Rev.* **73**, 766–791 (2008). 25324575 10.1177/000312240807300504 PMC4196718

[21] Jaffard, S., Seuret, S., Wendt, H., Leonarduzzi, R. & Abry, P. Multifractal formalisms for multivariate analysis. *Proc. Roy. Soc. A* **475**, 20190150 (2019). 31611713 10.1098/rspa.2019.0150 PMC6784393

[22] Lucas, C.-G., Abry, P., Wendt, H. & Didier, G. Epileptic seizure prediction from eigen-wavelet multivariate self-similarity analysis of multi-channel EEG signals. In *2023 31st European Signal Processing Conference (EUSIPCO)*, 970–974 (IEEE, 2023).

[23] Myint, S. W., Lam, N.S.-N. & Tyler, J. M. Wavelets for urban spatial feature discrimination. *Photogram. Eng. Remote Sens.* **70**, 803–812 (2004).

[24] Chen, Y. Derivation of the functional relations between fractal dimension of and shape indices of urban form. *Comput. Environ. Urban Syst.* **35**, 442–451 (2011).

[25] Lengyel, J., Roux, S., Sémécurbe, F., Jaffard, S. & Abry, P. Roughness and intermittency within metropolitan regions-application in three French conurbations. *Environ. Plan. B Urban Analyt. City Sci.* **50**, 600–620 (2023).

[26] Sémécurbe, F., Tannier, C. & Roux, S. G. Spatial distribution of human population in France: Exploring the modifiable areal unit problem using multifractal analysis. *Geogr. Anal.* **48**, 292–313 (2016).

[27] Salat, H., Murcio, R., Yano, K. & Arcaute, E. Uncovering inequality through multifractality of land prices: 1912 and contemporary kyoto. *PLoS ONE* **13**, e0196737 (2018). 29709024 10.1371/journal.pone.0196737 PMC5927455

[28] Hu, S., Cheng, Q., Wang, L. & Xie, S. Multifractal characterization of urban residential land price in space and time. *Appl. Geogr.* **34**, 161–170 (2012).

[29] Lengyel, J., Alvanides, S. & Friedrich, J. Modelling the interdependence of spatial scales in urban systems. *Environ. Plan. B Urban Analyt. City Sci.* **50**, 182–197 (2023).

[30] Lengyel, J., Roux, S., Abry, P., Sémécurbe, F. & Jaffard, S. Local multifractality in urban systems’ the case study of housing prices in the greater Paris region. *J. Phys. Complex.* **3**, 045005 (2022).

[31] Meneveau, C., Sreenivasan, K., Kailasnath, P. & Fan, M. Joint multifractal measures: Theory and applications to turbulence. *Phys. Rev. A* **41**, 894 (1990). 9903171 10.1103/physreva.41.894

[32] Jaffard, S. et al. Multivariate multifractal analysis. *Appl. Comput. Harmon. Anal.* **46**, 653–663 (2019).

[33] Wendt, H. et al. Assessing cross-dependencies using bivariate multifractal analysis. In *2018 IEEE International Conference on Acoustics, Speech and Signal Processing (ICASSP)*, 4514–4518 (IEEE, 2018).

[34] INSEE. L’institut national de la statistique et des Ãtudes Ãconomiques. https://www.insee.fr/fr/statistiques/6215138?sommaire=6215217.

[35] Massey, D. S. & Denton, N. A. Suburbanization and segregation in us metropolitan areas. *Am. J. Sociol.* **94**, 592–626 (1988).

[36] Lengyel, J. et al. Bivariate multifractal analysis for non-homogenous point processes, with application to geospatial data. In *2023 31st European Signal Processing Conference (EUSIPCO)*, 1778–1782 (IEEE, 2023).

[37] Roux, S. G. et al. Analysis of scale invariance in nonuniformly sampled processes. In *proceedings of the XXIXe colloque sur le Traitement du Signal et des Images. GRETSI, Grenoble, France* (2023).

[38] Tél, T., Fülöp, Á. & Vicsek, T. Determination of fractal dimensions for geometrical multifractals. *Physica A* **159**, 155–166 (1989).

[39] Song, Y.-Q., Liu, J.-L., Yu, Z.-G. & Li, B.-G. Multifractal analysis of weighted networks by a modified sandbox algorithm. *Sci. Rep.* **5**, 17628 (2015). 26634304 10.1038/srep17628 PMC4669438

[40] Whitcher, B., Guttorp, P. & Percival, D. B. Wavelet analysis of covariance with application to atmospheric time series. *J. Geophys. Res. Atmos.* **105**, 14941–14962 (2000).

[41] Wendt, H., Didier, G., Combrexelle, S. & Abry, P. Multivariate hadamard self-similarity: Testing fractal connectivity. *Physica D* **356**, 1–36 (2017).

[42] Jaffard, S. et al. p-Exponent and p-leaders, part I: Negative pointwise regularity. *Physica A* **448**, 300–318 (2016).

[43] Leonarduzzi, R. et al. p-Exponent and p-leaders, part II: Multifractal analysis. Relations to Detrended Fluctuation Analysis. *Physica A* **448**, 319–339 (2016).

[44] Iceland, J., Weinberg, D. H. & Steinmetz, E. *Racial and Ethnic Residential Segregation in the United States 1980–2000* Vol. 8 (Bureau of Census, 2002).

[45] Clerval, A. The spatial dynamics of gentrification in Paris: A synthesis map. *Cybergeo Eur. J. Geography* (2011).

[46] Pattaroni, L., Kaufmann, V. & Thomas, M.-P. The dynamics of multifaceted gentrification: A comparative analysis of the trajectories of six neighbourhoods in the île-de-France region. *Int. J. Urban Region. Res.* **36**, 1223–1241 (2012).

