# Supplementary material for: A bivariate multifractal analysis approach to understanding socio-spatial segregation dynamics
Source: Sci Rep. 2025 Feb 7;15:4610. doi: 10.1038/s41598-025-86024-9 (PMC11806034; doi:10.1038/s41598-025-86024-9)
Supplement: Supplementary file 1 — Supplementary Information. [file 41598_2025_86024_MOESM1_ESM.pdf]

Supporting information for the associated main article; A bivariate multifractal analysis approach to understanding socio-spatial segregation dynamics

J. Lengyel, S. Roux, O. Bonin, S. Jaffard, and P. Abry

Firstly, this supporting information contains five additional illustrations that facilitate interpretation and support the findings of the corresponding main article; Figure S1 shows the two original signals (on the 200×200 meter pixel-level), the share of under-18s and over-65s, used for the case studies, supplemented by equivalized disposable income (EDI) per capita and the share of poor households for comparison. The second supporting figure S2 displays the population density at $a = 2000$ meters for the case study of Paris and Lyon. Third, Figure S3 contains the original, i.e. not spatially smoothed, results for the classical segregation indices $\widetilde{D}(v,a)$ and $\widetilde{P}^{1.2}(v,a)$. Figure S4 compares the local and global scaling for the two classical segregation measures and the two bivariate multifractal parameters. Finally, Figure S5 shows the theoretical multifractal spectra for the two data sets in the two study regions and discusses their relationship to the univariate multifractal parameters derived in Figure 2 in the main article. The remaining part of the supporting information contains an overview of all calculations from the main article to support methodological comprehensiveness. The aim is to facilitate reproducibility with a brief overview of the equations and their exact inputs. All detailed explanations of their meaning and interpretations can be found in the main article. Similar to the main article, we use the abbreviations CS for classical segregation and MF for the multifractal parameters.

Methdological overview

In accordance with the main article: To ensure consistency between the two methodological sections, the superscript t stands for the total population, while i ($i = (1,2)$) represents the respective subgroup value. First the bisquare weighting function is obtained for any weighting distance a' as

$$w_{v,v'}(a') = \begin{cases} \left(1 - \left(d_{v,v'}/a'\right)^2\right)^2 \text{if } d_{v,v'} < a' \\ 0, \text{otherwise} \end{cases} \quad \text{with } d_{v,v'} = \sqrt{(x_{v'} - x_v)^2 + (y_{v'} - y_v)^2} \tag{S.1}$$

where the weights are normalized so that $\sum_{v'} w_{v,v'}(a') = 1$. For any variable $X(v,a)$, which jointly depends on the space $v = (x_v, y_v)$ and the scale a, we define the geographically weighted mean, as

$$\widetilde{M}_{X,a'}(v,a) = \sum_{v'} w_{v,v'}(a') X(v',a), \tag{S.2}$$

the geographically weighted variance

$$\widetilde{V}_{X,a'}(v,a) = \sum_{v'} w_{v,v'}(a') \left((X(v',a) - \widetilde{M}_{X,a'}(v,a) \right)^2 \tag{S.3}$$

as well as the global mean:

$$\overline{X}(a) = \frac{1}{N_v} \sum_{v} X(v,a) \tag{S.4}$$

where N_v is the total number of available sites v. In the following parts of this supporting material, we will use the notation $n_v(a)$ to refer to the number of available sites v in a ball of radius a. For data $X(v)$ only depending on space, we simply write the above-defined parameters as $\widetilde{M}_{X,a'}(v)$, $\widetilde{V}_{X,a'}(v)$ and $\overline{X}$ removing the extra variable a.

Classical segregation The dataset $\mathscr{S}(v, \kappa_v^i, \kappa_v^t)$ is defined by its geolocation $v = (x_v, y_v)$ with x_v the latitude and y_v longitude coordinate (the support), along with one or more associated values κ_v^i and κ_v^t (the marks). The population intensity within a ball of radius a is then expressed as $\widetilde{M}_{\kappa^t,a}(v)$ and $\widetilde{M}_{\kappa^i,a}(v)$ and the local ratio of these two parameters describes the preliminary component of the classical segregation measures discussed here as

$$\widetilde{\tau}^i(v,a) \quad = \quad \frac{\widetilde{M}_{\kappa^i,a}(v)}{\widetilde{M}_{\kappa^t,a}(v)}. \tag{S.5}$$

In their spatial and generalized form the *local ($\widetilde{D}(v,a)$) and global (D) dissimilarity*, along the *evenness dimension* of segregation, are defined as

$$\widetilde{D}(v,a) = \sum_i \frac{\kappa_v^t}{2IK^t} |\widetilde{\tau}^i(v,a) - \Pi^i| \quad \text{and} \quad D(a) = \sum_v \widetilde{D}(v,a) \tag{S.6}$$

where $I = \sum_i \Pi^i(1-\Pi^i)$ and $\Pi^i = \frac{\sum_v \kappa_v^i}{K^t}$ is the global *aspatial* proportion of the population group i and K^i and K^t are the corresponding *aspatial* subgroup $K^i = \sum_v \kappa_v^i$ and total population, $K^t = \sum_v \kappa_v^t$, of the entire study area. Regarding the second *exposure dimension of segregation*, the local and global two-group exposure indices ($i = (1,2)$) are expressed as follows;

$$\widetilde{P}^{1,2}(v,a) = \frac{\kappa_v^1}{K^1}\widetilde{\tau}^2(v,a) \quad \text{and} \quad P^{1,2}(a) = \sum_v \widetilde{P}^{1,2}(v,a). \tag{S.7}$$

Multifractal analysis For a better overview, we will first recapitulate the calculation of the multifractal parameters from the main article and then define their global scaling. Parameters are obtained within a local environment L, i.e., $a' = L$ in eqs. S.1, S.2, S.3.

Scale-free analysis If $n_v(a)$ is the number of data points located in a ball centered at v and within a radius a, then the multiscale quantity is derived as

$$\begin{cases} \gamma_v^i & = \frac{\kappa_v^i}{\kappa_v^t} \\ O^i(v,a) & = \frac{1}{n_v(a)} \sum\limits_{v',\, d_{v,v'} \leq a} \gamma_{v'}^i \\ O^i(v,\sqrt{2}a) & = \frac{1}{n_v(a,\sqrt{2}a)} \sum\limits_{v',\, a<d_{v,v'} \leq \sqrt{2}a} \gamma_{v'}^i \\ U^i(v,a) & = O^i(v,a) - O^i(v,\sqrt{2}a), \end{cases} \tag{S.8}$$

where $n_v(a,\sqrt{2}a)$ is the number of observations v in a ring between the distances a and $\sqrt{2}a$[1]. The univariate multiscale parameter is obtained according to

$$C_1^i(v,a,L) \quad = \quad \widetilde{M}_{\log|U^i|,L}(v,a), \; i = (1,2), \tag{S.9}$$

$$C_1^i(v,a,L) \quad \sim_{a\to 0} \quad c_1^i(v,L)\log(a) + k_1^i(v), \tag{S.10}$$

and the bivariate parameter as

$$\rho_{ss}(v,a,L) = \frac{\widetilde{M}_{U^1U^2,L}(v,a) - \widetilde{M}_{U^1,L}(v,a)\widetilde{M}_{U^2,L}(v,a)}{\sqrt{\widetilde{V}_{U^1,L}(v,a)\widetilde{V}_{U^2,L}(v,a)}}. \tag{S.11}$$

Multifractal analysis The multiscale quantity used here is expressed by the wavelet p-leader ($p = 2$), as

$$Q^i(v,a,p) \quad = \quad \left(\frac{1}{n_v(a)} \sum_{v',\, d_{v,v'} \leq a} |U^i(v',a)|^p \right)^{1/p}. \tag{S.12}$$

The univariate multifractal parameter is defined according to

$$C_2^i(v,a,L) \quad = \quad \widetilde{V}_{\log|Q^i|,L}(v,a), \; i = (1,2), \tag{S.13}$$

$$C_2^i(v,a,L) \quad \sim_{a\to 0} \quad c_2^i(v,L)\log(a) + k_2^i(v), \tag{S.14}$$

and the corresponding bivariate multifractal parameter as

$$\rho_{mf}(v,a,L) = \frac{\widetilde{M}_{C_2^1C_2^2,L}(v,a) - \widetilde{M}_{C_2^1,L}(v,a)\widetilde{M}_{C_2^2,L}(v,a)}{\sqrt{\widetilde{V}_{C_2^1,L}(v,a)\widetilde{V}_{C_2^2,L}(v,a)}}. \tag{S.15}$$

Practically, $c_1^i(v,L)$ and $c_2^i(v,L)$ are estimated by performing linear regressions for each point $v(x_v, y_v)$ across a range of scales $a_{min} \leq a \leq a_{max} = L$.

Global scaling To evaluate global scaling characteristics in the multifractal analysis context, the mean values over all estimation points v are calculated. If N_v is the total number of all sites v, then,

$$\begin{aligned} \overline{C}_1^i(v,a,L) &= \frac{1}{N_v}\sum_v C_1^i(v,a,L) \\ \overline{C}_2^i(v,a,L) &= \frac{1}{N_v}\sum_v C_2^i(v,a,L) \\ \overline{\rho_{ss}}(v,a,L) &= \frac{1}{N_v}\sum_v \rho_{ss}(v,a,L) \\ \overline{\rho_{mf}}(v,a,L) &= \frac{1}{N_v}\sum_v \rho_{mf}(v,a,L). \end{aligned} \tag{S.16}$$

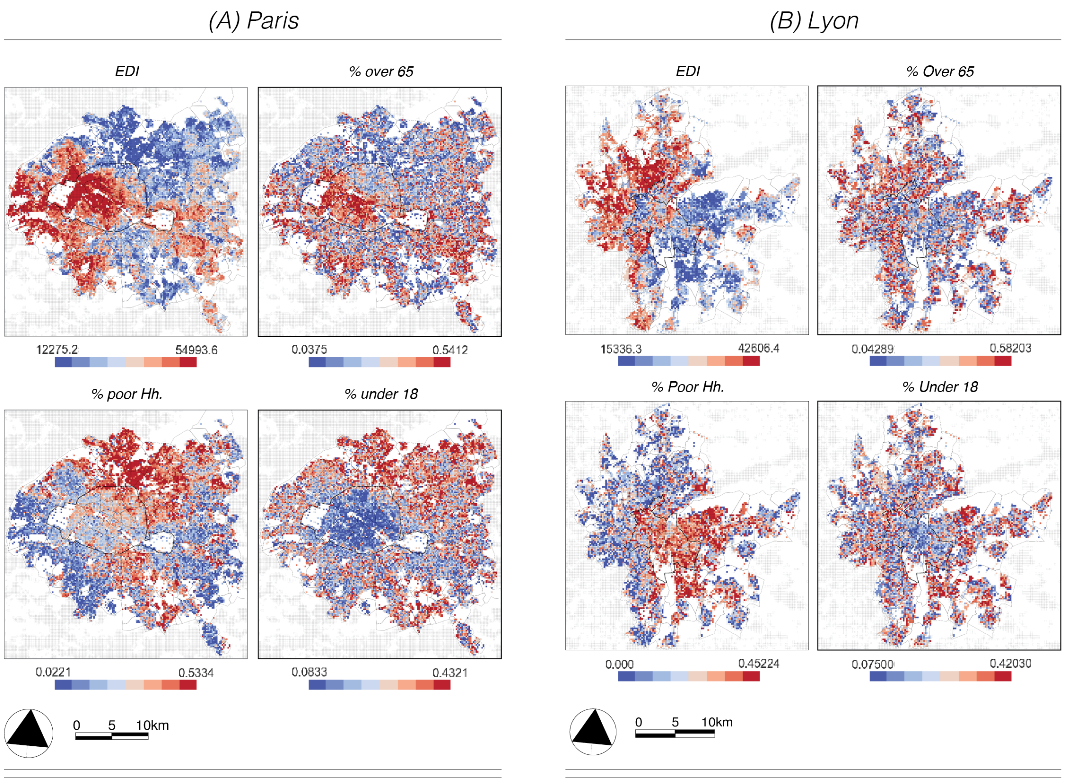

Fig S1. Original data at the 200×200 **meter pixel level**; the share of people under 18 and over 65 and their relationship to socioeconomic indicators: the proportion of poor households (H.h.) and the equivalized disposable income (EDI) per capita (p.c.). Data source; INSEE[2].

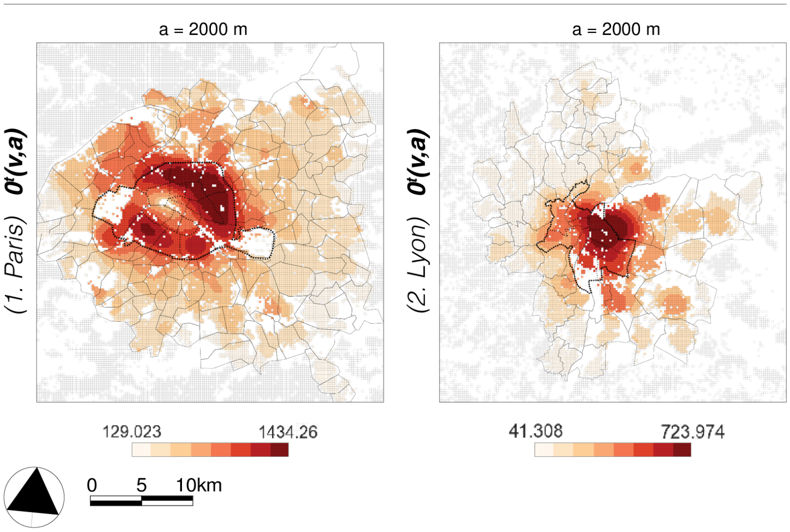

Fig S2. Population density; for the metropolitan region of Paris (1) and Lyon (2) at $a = 2000$ meters using the signal κ_v^t or the total number of individuals.

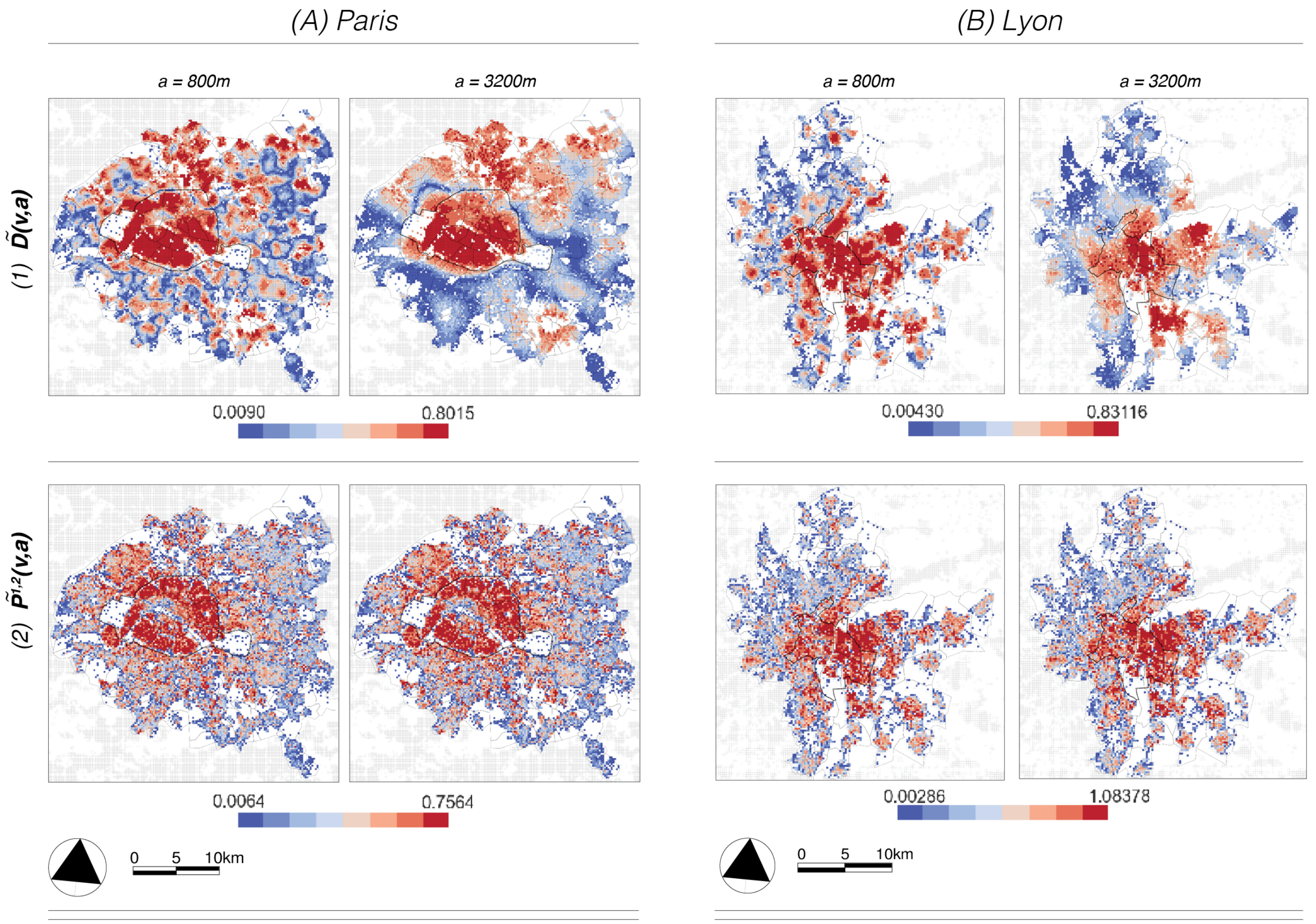

Fig S3. Original results for the classical segregation analysis; i.e. not spatially smoothed results for the indices $\widetilde{D}(v,a)$ (1) and $\widetilde{P}^{1.2}(v,a)$ (2). For better readability, we only show the results for the smallest ($a = 800$) and largest ($a = 3200$) observed scales. The values are multiplied by the constant N_v - the total number of observations v - to increase the legibility of legends.

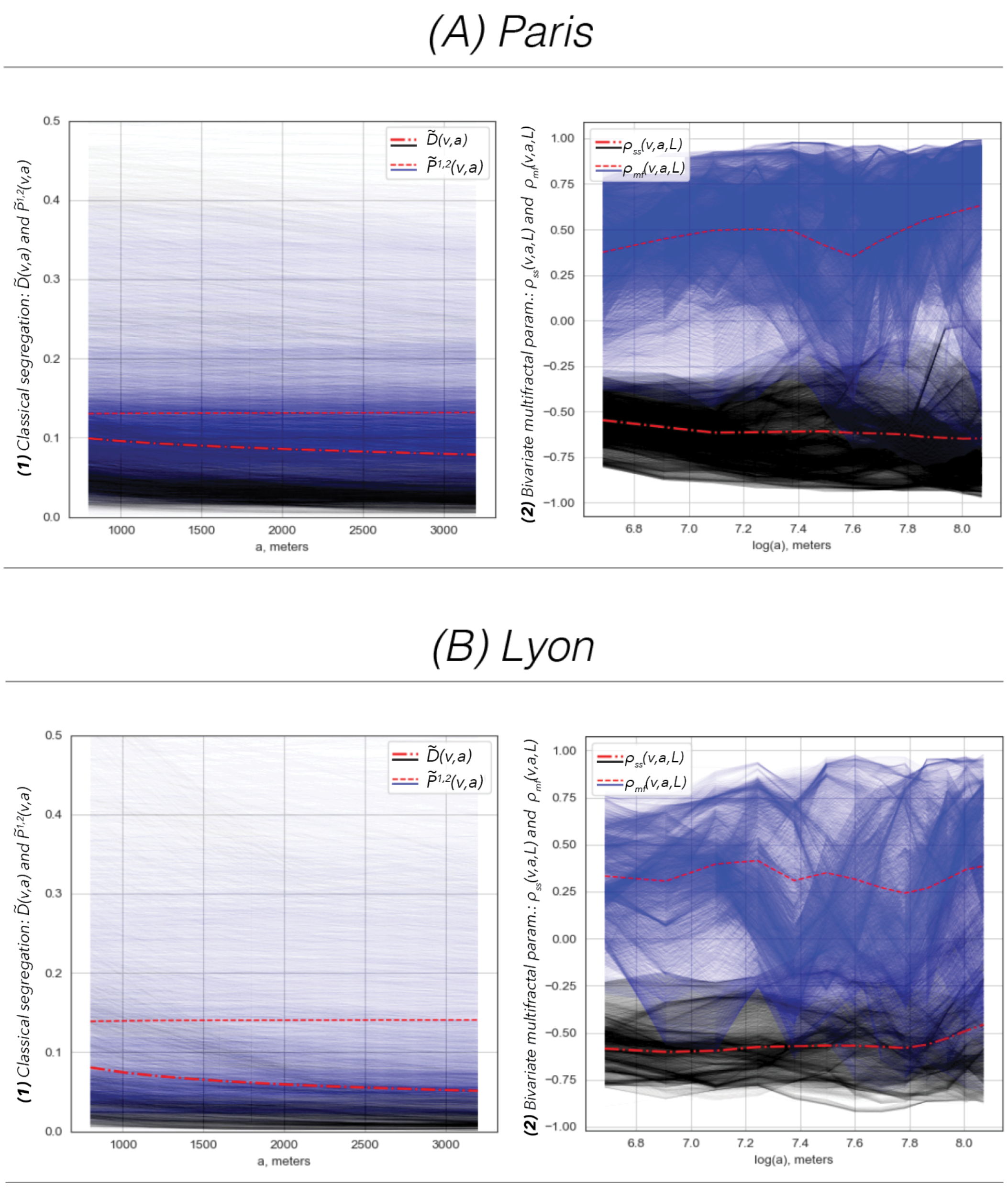

Fig S4. Bivariate CS and MF parameters as functions of the logarithm of the scales, in the Paris (A) and Lyon (B) regions; (1) Scaling at each original point v for the CS indices $\widetilde{D}(v,a)$ (black) and $\widetilde{P}^{1.2}(v,a)$ (blue). (2) Local scaling for the bivariate multifractal parameters $\rho_{ss}(v,a,L)$ (black) and $\rho_{mf}(v,a,L)$ (blue). The progression across scales a in a local environment L (2, black and blue curves) for the bivariate MF parameters is visibly more pronounced and irregular. The corresponding global scaling for the entire study regions in average are marked with the red curves (see also eqs. S.16). Note that the spatial representation of the results in this figure can be found in the main article in Figure 3 for the CS and in Figure 6 for the MF parameters.

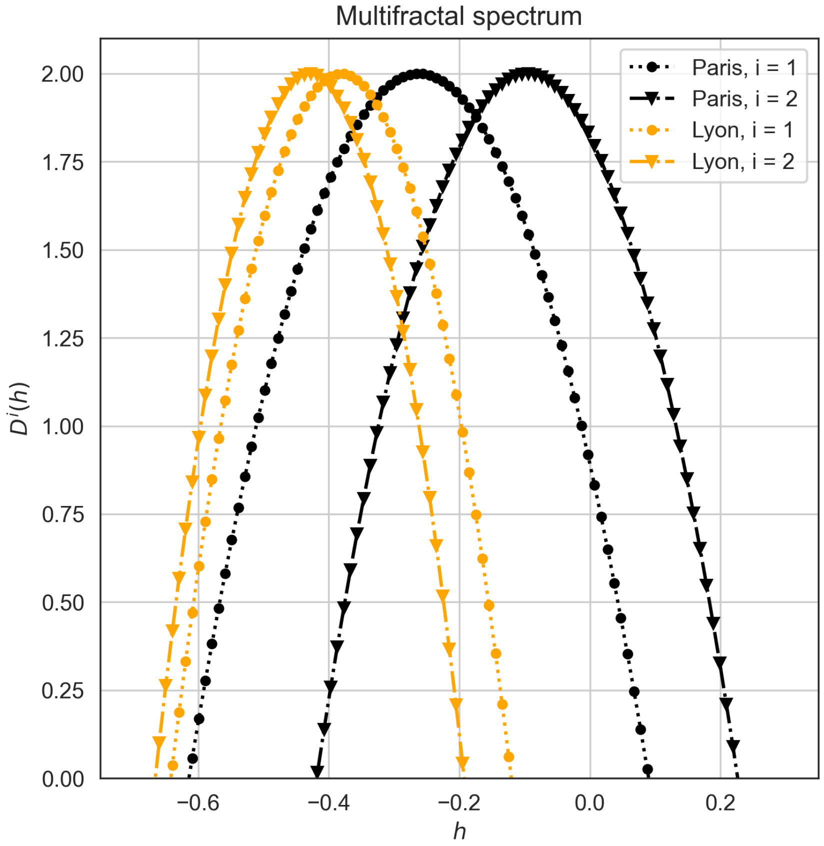

Fig S5. Univariate multifractal spectrum for the data sets in the Paris (black) and Lyon (orange) regions; ($i = 1$, circle) the share of people under 18 and ($i = 2$, triangle) over 65. Both the spatial dependencies and the degree of multifractality (width of the spectra) are more pronounced for the populations observed in Paris. Note that the point on the x-axis where the theoretical spectra reach their maximum is exactly at the derived $c_1^i(v,L)$ values in Figure 2 of the main article. The spectra are estimated according to $D^i(h) = d - (h - c_1^i(v,L))^2/(2c_2^i(v,L))^3$, with a theoretical collection of Hölder exponents h.

References

1. Lengyel, J., Roux, S., Sémécurbe, F., Jaffard, S. & Abry, P. Roughness and intermittency within metropolitan regions-application in three french conurbations. Environ. Plan. B: Urban Anal. City Sci. **50**, 600–620 (2023).
2. INSEE. L'institut national de la statistique et des études économiques, https://www.insee.fr/fr/statistiques/6215138?sommaire=6215217.
3. Wendt, H., Roux, S. G., Jaffard, S. & Abry, P. Wavelet leaders and bootstrap for multifractal analysis of images. Signal Process. **89**, 1100–1114 (2009).
